# Utility of 6-aza-2-thiothymine in the synthesis of novel [1,2,4]triazolo[4,3-*b*][1,2,4]triazin-7-one derivatives: synthesis, structure elucidation, molecular docking and *in vitro* anti-lung cancer activity†

**DOI:** 10.1039/d4ra08958h

**Published:** 2025-02-26

**Authors:** Monica G. Kamel, Farid M. Sroor, Khaled Mahmoud, Heba I. Shafey, Hamdi M. Hassaneen, Laure Vendier

**Affiliations:** a Department of Chemistry, Faculty of Science, Cairo University Giza Egypt hamdi_251@yahoo.com hhassaneen@sci.cu.edu.eg; b Organometallic and Organometalloid Chemistry Department, National Research Centre 12622 Cairo Egypt faridsroor@gmx.de fm.sroor@nrc.sci.eg; c Pharmacognosy Department, Pharmaceutical and Drug Industry Institute, National Research Centre 12622-Dokki Egypt; d Cell Biology Department, National Research Centre 12622-Dokki Egypt; e LCC-CNRS, Université de Toulouse, CNRS, UPS Toulouse France

## Abstract

Using 6-aza-2-thiothymine (ATT) as a suitable precursor, a novel series of [1,2,4]triazolo[4,3-*b*][1,2,4]triazin-7-one derivatives (7a–j) was prepared by refluxing 6-methyl-3-thioxo-3,4-dihydro-1,2,4-triazin-5(2*H*)-one (3) with hydrazonoyl halides (1a–j) in chloroform in the presence of triethylamine. The structures of the newly synthesized compounds 7a–j were confirmed using spectral data, elemental analyses, and single-crystal X-ray diffraction results. All the synthesized triazolotriazin-7-one derivatives (7a–j) were evaluated as *in vitro* anti-cancer agents against PC3 (prostate cell line), A549 (lung carcinoma), PACA2 (pancreatic cancer cell line) and BJ1 (normal skin fibroblast) cell lines using MTT assay. Compounds 7a and 7g showed greater efficacy and low IC_50_ values (36.6 and 40.1 μM, respectively) compared to the reference drug, which exhibited an IC_50_ value of 43.8 μM on the lung cell line, and demonstrated safe mortality effect on the normal cell line (BJ1) with cytotoxicity percentages of 3.5% and 2.8%, respectively. These compounds (7a and 7g) were the most active compounds of the synthesized triazolotriazin-7-one derivatives (7a–j). They were further investigated to ascertain their mechanism of action using DNA fragmentation, DNA damage and gene expression (BCL-2, BAX, and p53 genes). Results indicated a significant increase in the expression levels of BCL-2 and a reduction in the expression of p53 and BAX genes in negative lung cancer cell lines. However, the treatment of negative cell lines with 7g improved the expression of the tested genes to a greater extent than that with 7a. Additionally, the DNA damage and DNA fragmentation levels were significantly elevated in the lung cancer cell line samples treated with 7a much more than 7g. Molecular docking was employed to explore the potential interactions between the most active compounds (7a and 7g) and two key enzymes, human 3-phosphoglycerate dehydrogenase (PHGDH) and phosphoserine aminotransferase (PSAT1), which play vital roles in the progression of lung cancer.

## Abstract 2

Novel triazolo[4,3-*b*][1,2,4]triazin-7-one derivatives (7a–j) were synthesized and tested against PC3, A549, PACA2 and BJ1 cell lines. 7a and 7g were investigated using DNA fragmentation, DNA damage and gene expression and molecular docking studies.

## Graphical abstract

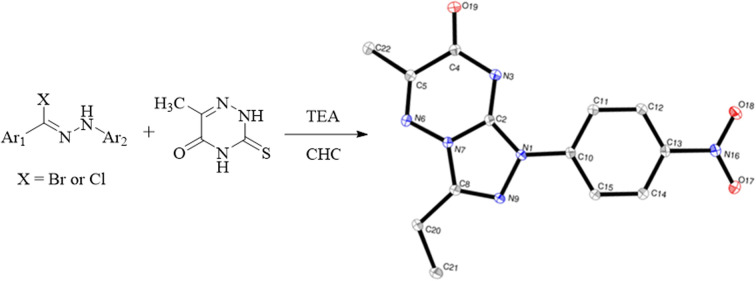

## Introduction

Triazolotriazine moiety has attracted significant interest in medicinal chemistry owing to its unusual structural and electrical characteristics, which improve the efficacy of therapeutic candidates.^1–4^ Its core is useful in the development of nucleoside analogs and other therapeutic medicines because it shows enhanced lipophilicity and improved binding interactions with biological targets.^1,5^ Its incorporation in potential drug candidates has been demonstrated to improve antiviral and anti-cancer properties, mainly by increasing the drug's potency and selectivity against particular enzymes and receptors.^6,7^ Furthermore, triazolotriazine moiety can improve the pharmacokinetic profiles of drugs and stabilize nucleic acid structures, enhancing bioavailability and lowering toxicity, respectively.^7,8^ The significance of triazolotriazine derivatives in the synthesis of novel therapeutic agents highlights their potential for treating a range of diseases, such as cancer and viral infections.

Lung cancer continues to be one of the most widespread and lethal types of cancer worldwide, causing serious problems to public health.^9,10^ Often discovered at an advanced stage, this disease is characterized by the uncontrolled proliferation of aberrant cells in the lungs, which results in poor prognosis and limited therapeutic options.^11^ The majority of lung cancer cases are caused by smoking, which is one of the main causes of the disease; however, risk factors for non-smokers include genetic predisposition, radon exposure, and environmental pollution.^12^ Moreover, the heterogeneity of lung cancer, which results in different subtypes displaying unique molecular profiles, makes treatment plans more challenging to implement and calls for personalized approaches.^13^ Targeted therapy and immunotherapy have made significant advances in lung cancer. However, issues including drug resistance, treatment expenses, and the necessity for early diagnostic technologies make it difficult to manage patients effectively and increase survival rates.^14,15^ Addressing these challenges requires an integrated strategy that includes prevention, early diagnosis, and the development of innovative therapeutic strategies, including discovering new drugs to improve the outcomes for patients with lung cancer.

Given the above information, the purpose of this study was to design and synthesize novel triazolo[4,3-*b*][1,2,4]triazin-7-one derivatives (7a–j) as the triazolotriazine family using 6-aza-2-thiothymine (ATT) as a suitable precursor and evaluate their anti-cancer activity against various cancer cell lines, including the PC3 (Prostate cell line), A549 (Lung carcinoma), PACA2 (Pancreatic cancer cell line) and BJ1 (normal skin fibroblast). The synthesis of the triazolo[4,3-*b*][1,2,4]triazin-7-one derivatives was of special interest since these compounds were not investigated in previous studies. The most promising compounds (7a and 7g) were examined in further studies to determine their mechanism of action using DNA fragmentation, DNA damage and gene expression (BCL-2, BAX, and p53 genes) as well as molecular docking.

## Results and discussion

### Chemistry

**Scheme 1:**
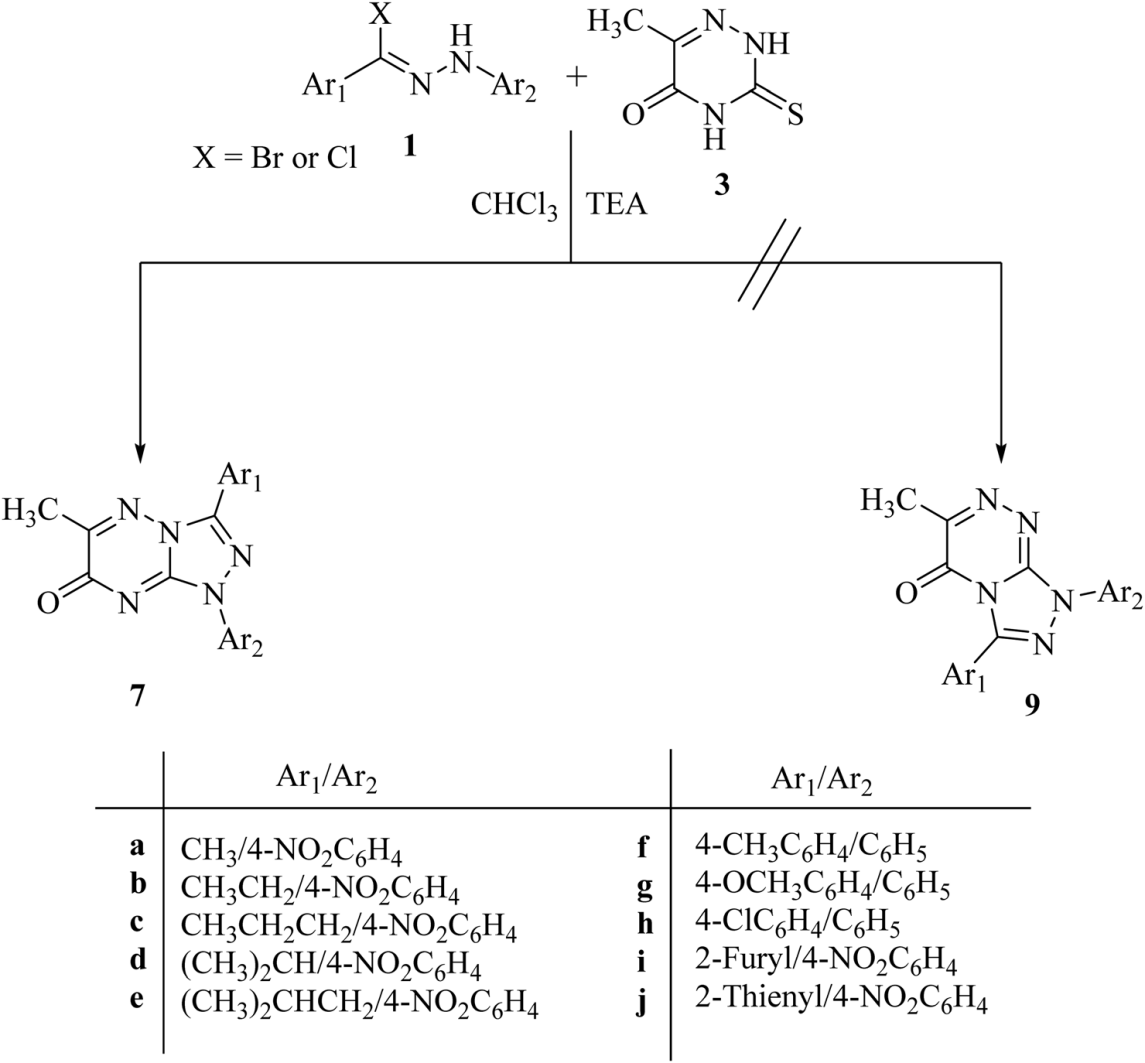
Synthesis of [1,2,4]triazolo[4,3-*b*][1,2,4]triazin-7-one derivatives 7a–j.

**Fig. 1:**
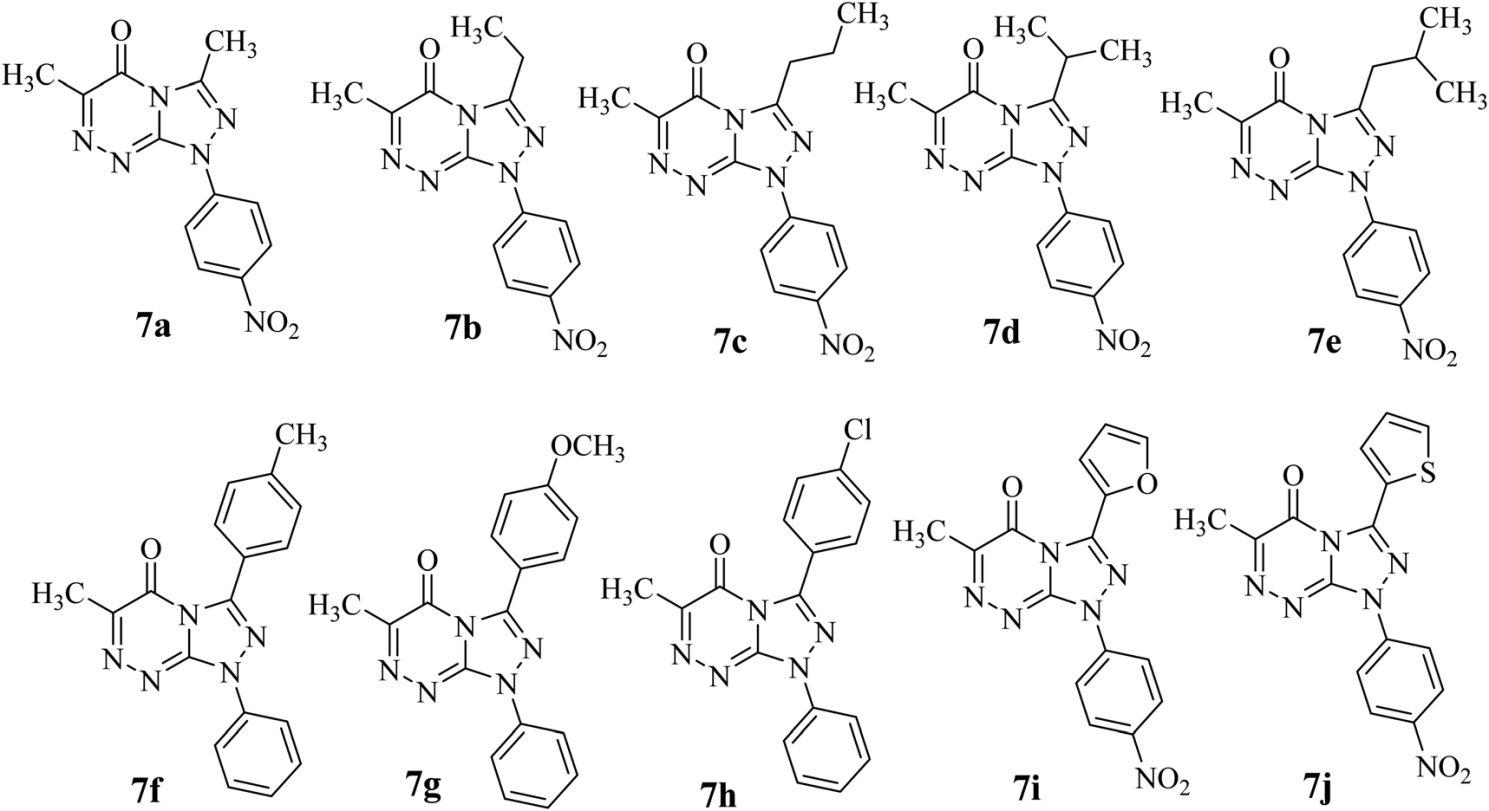
Chemical structures of all the synthesized [1,2,4]triazolo[4,3-*b*][1,2,4]triazin-7-one derivatives (7a–j).

**Scheme 2:**
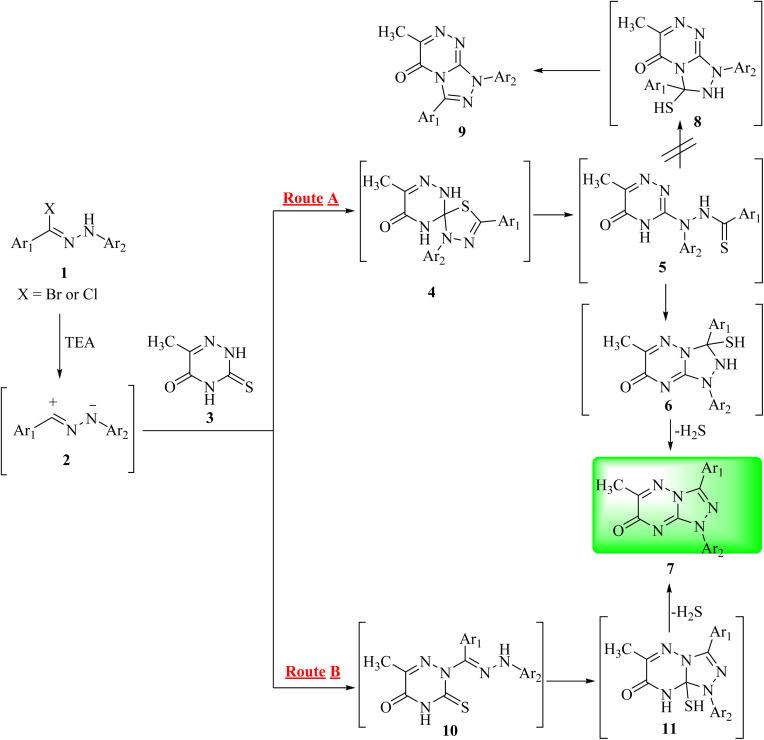
Plausible mechanism of the synthesis of [1,2,4]triazolo[4,3-*b*][1,2,4]triazin-7-one derivatives (7a–j).

The reaction of 6-methyl-3-thioxo-3,4-dihydro-1,2,4-triazin-5(2*H*)-one (3)^16^ with hydrazonoyl halides (1a–j) in refluxing chloroform in the presence of triethylamine yielded a single product in each case (7a–j), as shown in Scheme 1 and Fig. 1. In order to clarify the preceding results, Scheme 2 proposes two plausible mechanistic sequences. In the first sequence (route A in Scheme 2), nitrilimines (2), which are generated *in situ* by the base-catalyzed dehydrohalogenation of hydrazonoyl halides (1),^17–21^ are thought to undergo 1,3-dipolar cycloaddition with the C=S double bond of triazinethione (3), resulting in the spiro intermediate 4, which is then subjected to a base-catalyzed ring cleavage to yield thiohydrazide (5). Subsequent intramolecular cyclization of 5 may result in two distinct heterocyclic intermediates, 6 and 8, which quickly lose hydrogen sulfide to form triazolo[4,3-*b*][1,2,4]triazin-7-one (7) or triazolo[3,4-*c*][1,2,4]triazin-5-one (9), respectively. In the intermediate 5, the less nucleophilic nitrogen (N-4) could lead to a preferential ring closure to form [1,2,4]triazolo[4,3-*b*][1,2,4]triazin-7-one (7) owing to the electronic effect of the carbonyl group.^22^ As an alternative, the reaction could include primary 1,3-addition, which results in amidrazones (10) that cyclize to give intermediate 11 and then compound 7 by the loss of hydrogen sulfide, as shown in route B in Scheme 2. It is suggested that route A is more likely to occur considering the following factors: (1) a C=S double bond appears to be a more reactive dipolarophile toward different 1,3-dipoles, (2) amidrazones of type 10 are known to be stable, but despite this, every attempt to separate them from the reaction mixtures was unsuccessful; (3) triazine-3-thione derivatives are mainly found in thione structures.^23^

**Fig. 2:**
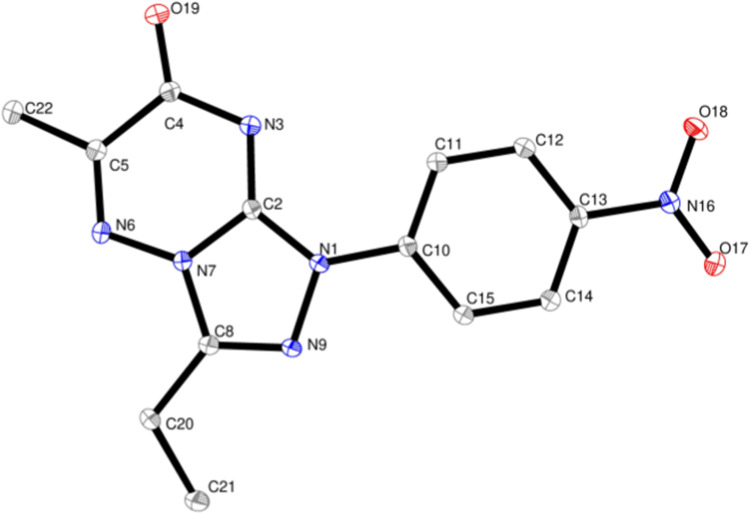
Single-crystal structure of 3-ethyl-6-methyl-1-(4-nitrophenyl)-[1,2,4]triazolo[4,3-*b*][1,2,4]triazin-7(1*H*)-one (7b). H atoms are omitted for clarity. Selected bond lengths [Å]: N7–N6 1.3721(14), N9–N1 1.3969(14), N7–C2 1.3648(16), C2–N1 1.3634 (15), N3–C2 1.3119(16), C10–N1 1.4156(15), O19–C4 1.2236(16), C22–C5 1.4916(17), O17–N16 1.2289(15), O18–N16 1.2285(15). Selected bond angles [°]: C8–N9–N1 106.15(10), O17–N16–O18 123.50(11), N6–N7–C8 126.81(11), N6–N7–C2 123.68(11), C8–N7–C2 109.18(10), N9–N1–C2 110.77(10), N3–C2–N1 130.12(11).

The structures of the isolated products were identified by their elemental analyses and spectroscopic data (^1^H NMR and ^13^C NMR). Their structures were assigned to be 7 rather than the isomeric structure 9 (Scheme 1 and Fig. 1). For example, the ^1^H NMR spectrum of compound 7b showed the following signals: triplet at *δ* 1.48 corresponding to the methyl protons in CH_2_CH̲_3_, singlet at *δ* 2.45 corresponding to CH_3_ group protons, quartet at *δ* 3.03 corresponding to the methylene protons in CH̲_2_CH_3_ and pair of doublets at *δ* 8.30 and 8.45 corresponding to the protons of 4-NO_2_C_6_H_4_. Also, its ^13^C NMR spectrum showed 11 signals for asymmetric carbon atoms. Moreover, the single crystal X-ray structure of 7b confirmed the formation of a [1,2,4]triazolo[4,3-*b*][1,2,4]triazin-7-one scaffold (Table 1 and Fig. 2).

**Table 1:** Crystal structure data and details of the structure refinement for compound 7b

CCDC deposition number	2390251	*μ* (Cu K_α_)/mm^−1^	0.95
Chemical formula sum	C_13_H_13_N_6_O_3_	Crystal size/mm	0.19 × 0.18 × 0.14
Formula weight/g mol^−1^	301.28	*T*/K	100
Crystal color and shape	Orange, Bloc	*θ* rang/°	4.2–79.4
Crystal system	Monoclinic	Reflections collected	2840
Space group	*P*2_1_/*n*	*λ* (Å)	1.54184
Unit cell parameters			
*a* (Å)	8.0047 (3)	*F*000	628
*b* (Å)	21.1874 (6)	Extinction correction	None
*c* (Å)	8.0296 (3)	*R*_Int_	0.042
*α* (°)	90	Parameters/restraints	203/4
*β* (°)	104.314 (4)	*R*[*F*^2^ > 2*σ*(*F*^2^)]	0.043
*γ* (°)	90	*wR*(*F*^2^)	0.1294
Unit cell volume/Å^3^	1319.53 (9)	Goodness-of-fit on *F*^2^	0.9825
Molecules per cell *Z*	4	1-Sigma level	0.001
Calcd density *ρ*/g cm^−3^	1.516	Highest difference peak and hole/e Å^−3^	0.29/−0.64

As depicted in Table 1 and Fig. 2, single crystals containing 7b were found to be suitable for single-crystal X-ray diffraction. The crystal structure of compound 7b was deposited at Cambridge Crystallographic Data Centre under CCDC number 2390251. It was produced as orange crystals from a saturated DMF/acetonitrile solution at ambient temperature. Compound 7b forms a single molecule in the unit cell and crystallizes in the monoclinic cell with space group *P*2_1_/*n* (Table 1). The presence of the fused [1,2,4]triazolo[4,3-*b*][1,2,4]triazin-7-one scaffold was clearly elucidated by the crystal structure determination of 3-ethyl-6-methyl-1-(4-nitrophenyl)-[1,2,4]triazolo[4,3-*b*][1,2,4]triazin-7(1*H*)-one (7b) (Fig. 2). The selected examples of the bond lengths and angles of the [1,2,4]triazolo[4,3-*b*][1,2,4]triazin-7-one moiety, the distances of N7–N6 is 1.3721(14), N9–N1 is 1.3969(14), N7–C2 is 1.3648(16), C2–N1 is 1.3634 (15), N3–C2 is 1.3119(16), and C10–N1 is 1.4156(15), and the angles C8–N9–N1 is 106.15(10), O17–N16–O18 is 123.50(11), and N6–N7–C8 is 126.81(11), are in good agreement with those reported for 5-(4-bromophenyl)-4,6-dichloro-7-(2,4-dichlorophenyl)-7*H*-pyrrolo[2,3-*d*]pyrimidine, 4-amino-7-nitro-[1,2,4]triazolo[5,1-*c*][1,2,4]triazine-3-carbonitrile and 4,6-dichloro-7-(2,4-dichlorophenyl)-5-(3,4-dimethoxyphenyl)-7*H*-pyrrolo[2,3-*d*]pyrimidine.^3,24,25^ More selected bond lengths and angles are shown in Fig. 2 (see ESI†).

### Anti-cancer activity

#### *In vitro* cytotoxicity using MTT assay

Using specific human cancer cell lines, the anti-proliferative effectiveness of compounds 7a–j was evaluated in an *in vitro* assay. The anti-cancer activity of these compounds (7a–j) on various cancer cell lines, including PC3 (Prostate cell line), A549 (Lung carcinoma), PACA2 (Pancreatic cancer cell line) and BJ1 (normal skin fibroblast), was investigated in this study using the 3-(4,5-dimethylthiazol-2-yl)-2,5-diphenyltetrazolium bromide (MTT) test. Doxorubicin (Dox) was the standard medication used as a positive control.

In the initial screening trial, a single dosage concentration of 100 μg ml^−1^ was applied for 48 hours. The cytotoxicity of the treated cells was determined by calculating the cell death percentage (% mortality) as a percentage of untreated cells (Table 2). Against the lung cancer cell lines (A549), the results demonstrated that four compounds, 7a, 7e, 7f and 7g, had considerable cytotoxic activity (80.4, 86.3, 69.1, and 82.3%, respectively), while compounds 7b, 7d and 7j showed limited cytotoxic effects of 35.2, 33.3 and 32.8%, respectively (Table 2). In the case of the prostate cell line (PC3), all compounds showed limited anti-cancer activity with mortality in the range of 15.9–35.1%, as depicted in Table 2. All the synthesized triazolotriazin-7-one derivatives (7a–j) demonstrated a low cytotoxic effect on the pancreatic cancer cell line (PACA2), as shown in Table 2. Additionally, to confirm the safety of the most effective compounds against cancer cells, their cytotoxicity was assessed against normal cells (BJ1). As visualized in Table 1, the most promising compounds, 7a, 7e, 7f and 7g, were found to be safe on the normal cells (BJ1) up to a concentration of 100 μg ml^−1^ with cytotoxicity percentages 3.5%, 6.4%, 3.2%, and 2.8%, respectively.

**Table 2:** (%) Mortality of cancer and normal cell lines at 100 μg ml^−1^ of compounds 7a–j and IC_50_ (μM) values of the most active compounds

Compound	PC3 (IC_50_, μM)	A549 (IC_50_, μM)	PACA2 (IC_50_, μM)	BJ1
7a	28.2 ± 0.25	80.4 ± 0.66 (36.6 ± 0.33)	20.4 ± 0.11	3.5 ± 1.16
7b	24.5 ± 0.57	35.2 ± 1.22	3.6 ± 0.21	4.2 ± 0.98
7c	27.3 ± 1.20	22.9 ± 0.81	10.5 ± 0.61	1.6 ± 0.38
7d	17.9 ± 0.38	33.3 ± 0.22	11.5 ± 0.25	36.2 ± 1.11
7e	35.1 ± 1.11	86.3 ± 0.74 (**42.5** ± **0.27**)	8.6 ± 0.33	6.4 ± 0.85
7f	33.4 ± 1.18	69.1 ± 0.77	5.6 ± 0.11	3.2 ± 0.63
7g	14.6 ± 0.92	82.3 ± 0.82 (**40.1** ± **0.84**)	13.4 ± 1.15	2.8 ± 0.71
7h	24.2 ± 0.21	13.5 ± 0.33	21.3 ± 0.98	9.6 ± 0.93
7i	18.4 ± 1.14	14.2 ± 0.24	9.6 ± 1.33	2.9 ± 1.22
7j	15.9 ± 0.51	32.8 ± 0.54	7.2 ± 0.88	3.8 ± 0.77
Dox	100 (**43.78** ± **0.54**)	100 (**43.80** ± **0.17**)	100 (**52.06** ± **0.42**)	1.10 ± 0.52
Negative control	0	0	0	0

Based on the results of Table 2, we continued our investigation into the molecular mechanisms underlying the potent lethal impact of the most active compounds on lung cancer cells (A549). Compounds 7a, 7e, 7f, and 7g showed limited mortality and high safety on the normal cells. Hence, secondary screening was performed on these compounds to ascertain their IC_50_ values and selectivity index. Table 2 illustrates that compounds 7a, 7e, and 7g had greater efficacy (IC_50_ values of 36.6, 42.5, and 40.1 μM, respectively) in comparison to the reference drug with an IC_50_ of 43.8 μM. Accordingly, compounds 7a and 7g with low IC_50_ values (36.6 and 40.1 μM, respectively) and safe mortality effect on the normal cell line (BJ1) with cytotoxicity percentages of 3.5, and 2.8%, respectively, are the most active compounds of the synthesized triazolotriazin-7-one derivatives (7a–j). Thus, in order to explore the potential mode of action of compounds 7a and 7g as powerful anti-lung cancer agents, we selected them for further studies. We used comet, RT-PCR, and DNA fragmentation tests to examine the effects of these compounds at the gene, protein, and DNA levels.

#### Gene expression in lung cancer cell lines

**Fig. 3:**
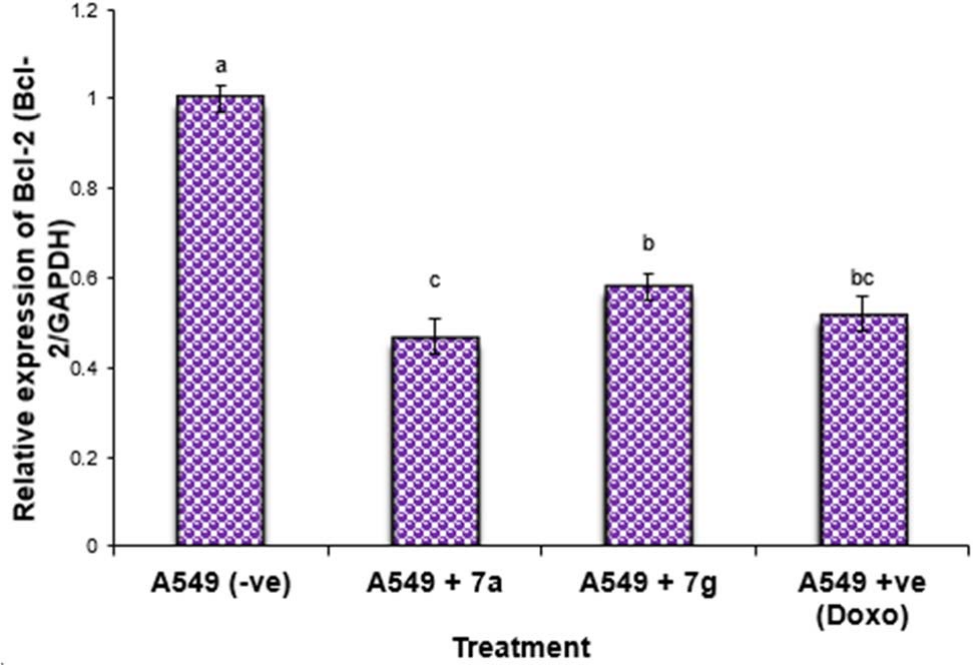
Alterations in the BCL-2 gene expression in lung cancer cell lines treated with 7a and 7g as well as Dox (as positive control). Data are presented as mean ± SEM. ^a, b, c^: Mean values within the tissue with unlike superscript letters were significantly different (*P* < 0.05).

**Fig. 4:**
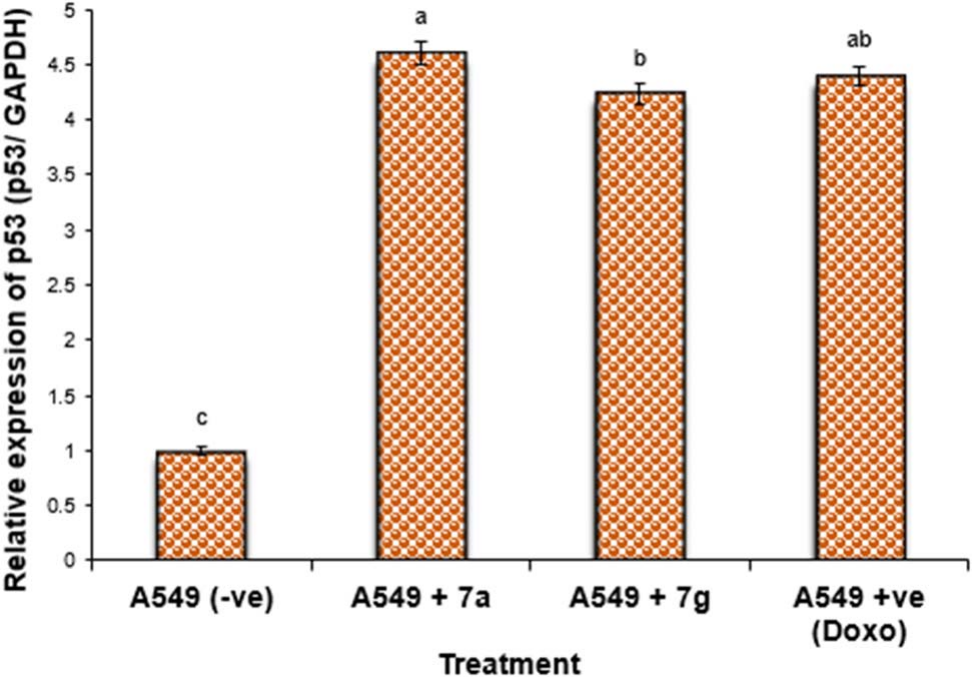
Alterations in the p53 gene expression in lung cancer cell lines treated with 7a and 7g as well as Dox. Data are presented as mean ± SEM. ^a, b, c^: Mean values within the tissue with unlike superscript letters were significantly different (*P* < 0.05).

**Fig. 5:**
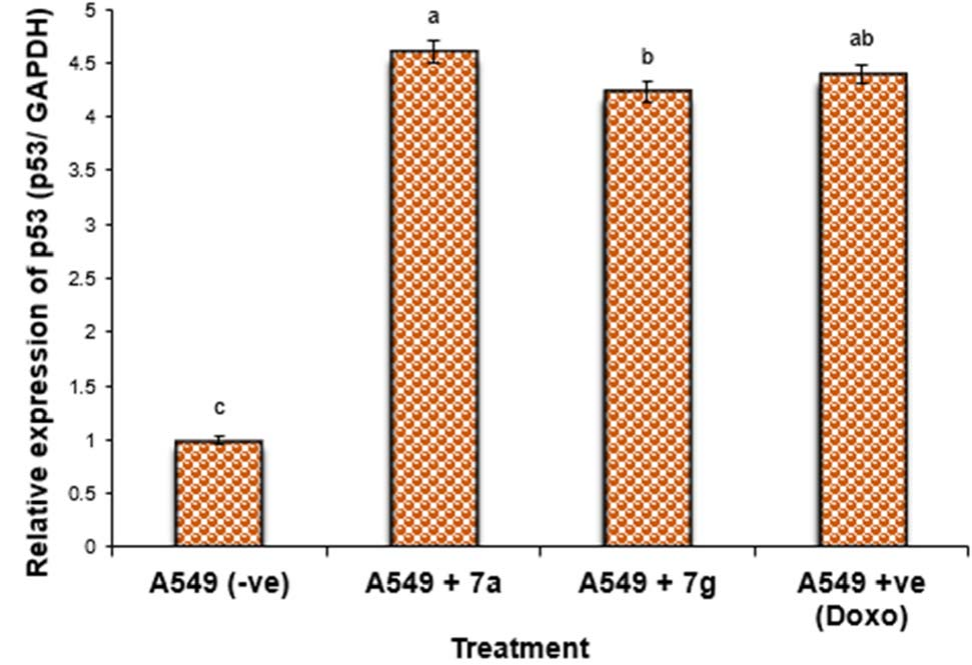
Alterations in the BAX gene expression in lung cancer cell lines treated with 7a and 7g as well as Dox. Data are presented as mean ± SEM. ^a, b, c^: Mean values within the tissue with unlike superscript letters were significantly different (*P* < 0.05).

The study of the expression of BCL-2, p53, and BAX in lung cancer cell lines treated with compounds 7a, 7g, and Dox is presented in Fig. 3–5. The results indicated a significant increase (*P* < 0.01) in the expression levels of the anti-apoptotic gene BCL-2 in negative samples of the lung cancer cell lines compared to the treated cell lines (Fig. 3).

Nonetheless, the expression levels of the pro-apoptotic genes (p53 and BAX) were dramatically diminished in the negative samples of lung cancer cell lines compared to the treated lung cancer cell lines (Fig. 4 and 5). The expression levels of the anti-apoptotic gene BCL-2 were diminished in descending order in the cancer cell lines treated with 7g, Dox, and subsequently 7a in comparison to the negative control cancer cell lines. The expression levels of the pro-apoptotic genes (p53 and BAX) were elevated in a sequential manner in the cancer cell line treated with 7g, followed by Dox, and subsequently 7a, in comparison to the negative control cancer cell lines.

The results indicated that the anti-cancer action of the examined drugs was most prominently detected in 7g, followed by Dox, and subsequently 7a. Compound 7a showed much greater efficacy against the tumor cell line compared to 7g.

#### DNA damage evaluation using the comet assay

**Fig. 6:**
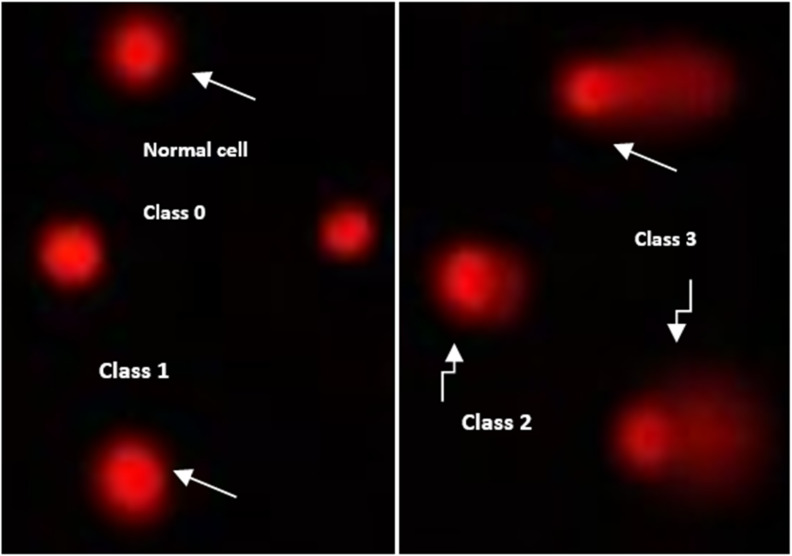
Visual score of normal DNAs (class 0) and damaged DNAs (class 1, class 2 and class 3) using comet assay in lung cancer cell lines.

The DNA damage in lung cancer cell lines was assessed using the comet test, as illustrated in Table 3 and Fig. 6. The findings indicated that the negative lung cancer cell lines demonstrated a notable reduction (*P* < 0.05) in DNA damage values (11.59 ± 0.65). However, the DNA damage levels were significantly elevated (*P* < 0.01) in the lung cancer cell line samples treated with 7a (28.181.25), Dox medication (23.861.11), and 7g (21.140.85) in comparison to the negative control.

**Table 3:** Visual score of the DNA damage in lung cancer cell lines treated with 7a and 7g

Treatment	No. of samples	No. of cells		Class^b^				DNA damaged cells% (mean ± SEM)
		Analyzed^a^	Comets	0	1	2	3	
A549 (−ve)	4	440	51	389	35	12	4	11.59 ± 0.65^c^
A549 + 7a	4	440	124	316	37	45	42	28.18 ± 1.25^a^
A549 + 7g	4	440	93	347	32	38	23	21.14 ± 0.85^b^
A549 + Dox	4	440	105	335	34	41	30	23.86 ± 1.11^b^

a Number of cells examined per group.

b Class 0 = no tail; 1 = tail length < diameter of the nucleus; 2 = tail length between 1× and 2× the diameter of the nucleus; and 3 = tail length > 2× the diameter of the nucleus.

#### Measurement of DNA fragmentation in lung cancer cell lines

**Fig. 7:**
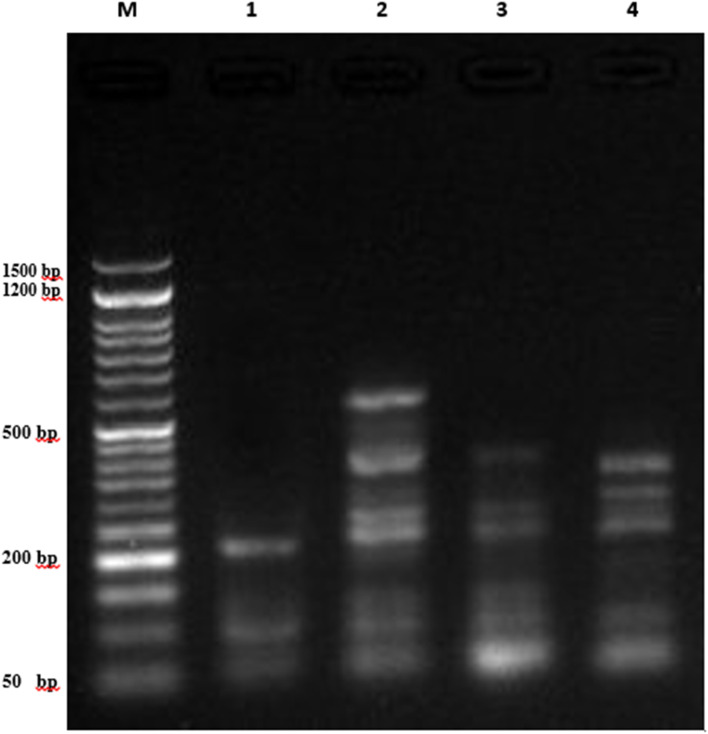
DNA fragmentation detected with agarose gel in A549 cancer cell lines exposed to different compounds. M represents the DNA marker, Lane 1 represents the negative control of A549, Lane 2 represents A549 treated with 7a, Lane 3 represents A549 treated with 7g, and Lane 4 represents A549 treated with Dox.

**Fig. 8:**
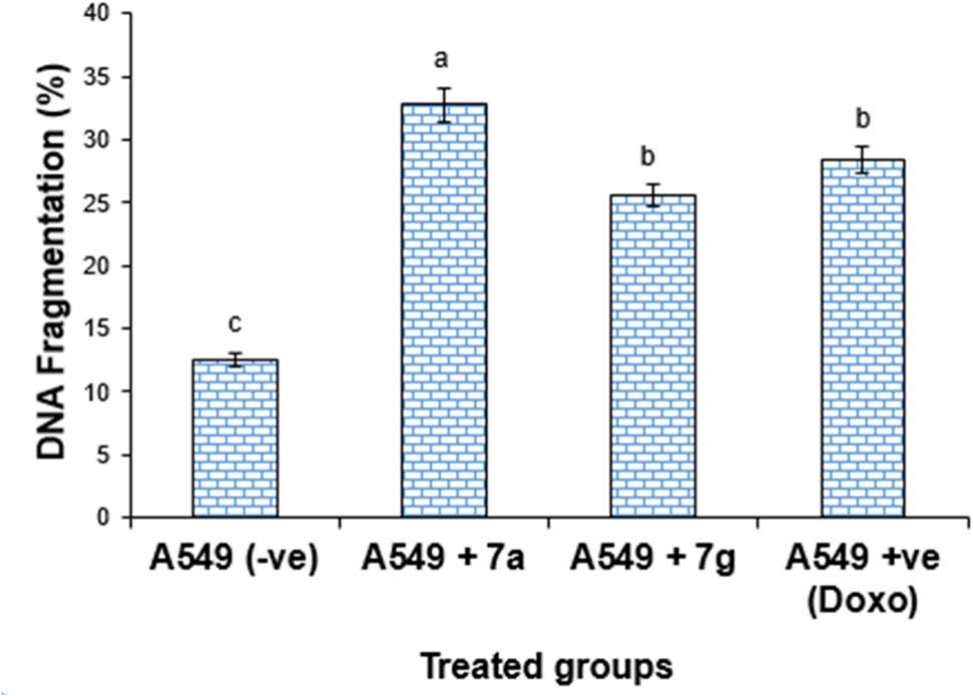
DNA fragmentation detected in the lung cancer cell lines (A549) treated with different compounds 7a, 7g and Dox. Mean values with different superscripts (^a, b, c^) between treatments in the same column are significantly different at *P* < 0.05.

The DNA fragmentation evaluation of compounds 7a and 7g against the lung cancer cell is depicted in Fig. 7 and 8 as well as Table 4. However, the DNA fragmentation values increased significantly (*P* < 0.01) in the lung cancer cell line samples treated with 7a (32.78 ± 1.35), doxorubicin (28.38 ± 1.06) and 7g (25.57 ± 0.94) compared with the negative control cancer cell lines (12.54 ± 0.45) (Table 5).

**Table 4:** DNA fragmentation detected in the lung cancer cell lines in different treatment groups^a^

Treatment	DNA fragmentation% (*M* ± SEM)	Change	Inhibition%
A549 (−ve)	12.54 ± 0.45^c^	0.00	0.00
A549 + 7a	32.78 ± 1.35^a^	20.24	27.78
A549 + 7g	25.57 ± 0.94^b^	13.03	17.74
A549 + Dox	28.38 ± 1.06^b^	15.84	19.69

a Means with different superscripts (^a, b, c^) between groups in the same column are significantly different at *P* < 0.05.

**Table 5:** Molecular docking results of the most active compounds 7a, 7g, and the co-crystalline ligand within the active pocket of 3-phosphoglycerate dehydrogenase (PHGDH) and phosphoserine aminotransferase (PSAT1)

Comp. no.	Score kcal mol^−1^	Moieties from compounds	Amino acid residues	Type of interaction, distance Å
**3-Phosphoglycerate dehydrogenase (PDB ID: 2G76)**				
NAD	−7.7	CO	Thr77, Arg235	H-bonds 3.12, 2.99
		O–P=O	His205, Ser102, Arg154, Ile155, Gly156	H-bonds 3.12, 3.22, 3.25, 3.20, 2.86, 3.40
		NH_2_, N	Ser211, Asp174	H-bonds 3.03, 3.37
		Phenyl ring	Arg235, Pro207	Alkyl
		CH_2_	His205	Carbon–H bond
7a	−7.8	NO_2_, N atoms of triazolo-triazine moiety	Ser211, His205, Asp174	H-bonds, 3.09, 2.84, 3.13
		Phenyl and triazine rings	Pro175, Pro207, Thr206	pi–alkyl, pi-sigma
		Triazole ring	Asp174	pi–anion
			Ile176, Gly153, Arg154	van der Walls
7g	−8.6	CO	Gly78	H-bond 2.33
		Phenyl and triazolo-triazine rings	Thr77, Ile155, Ala234, Pro207, Arg235	pi–donor H-bond, pi–alkyl

**Phosphoserine aminotransferase (PDB ID: 7T7J)**				
PLP	−6.5	CO, OH, O–P=O, N	Tr153, Lys198, Arg77, Gln197, Gly75, Asp174	H-bond 3.00, 2.72, 3.01, 2.98, 2.26, 2.85, 3.59
		P=O	Gly75	Carbon–hydrogen bond
			Trp102	van der Waals
7a	−7.4	NO_2_	Ser177, Ser176, Thr153	H-bonds 3.23, 3.18, 2.93, 3.49
		NO, phenyl and triazolo-triazine rings	Trp102, Gln197, Arg77	Carbon hydrogen bond, pi–donor hydrogen bond, pi–pi stacked, pi–alkyl interactions
			Cys149	van der Waals
7g	−8.5	CO, N atom of triazine ring	Lys198, Trp102, Gly10	H-bond 3.19, 3.01, 3.09
		Phenyl and 4-chloro phenyl rings	Pro11, Phe80, Trp102, Arg77	pi–pi stacked, pi–alkyl
		Triazole ring	Lys198	pi–cation
			Gln197	van der Waals

#### Molecular docking

Molecular docking is a valuable computational technique for assessing the binding interaction between a ligand and the active site of an enzyme or receptor. In this study, molecular docking was employed to explore the potential interactions between the most active compounds (7a and 7g) and two key enzymes, human 3-phosphoglycerate dehydrogenase (PHGDH) and phosphoserine aminotransferase (PSAT1), which play vital roles in the progression of lung cancer.

**Fig. 9:**
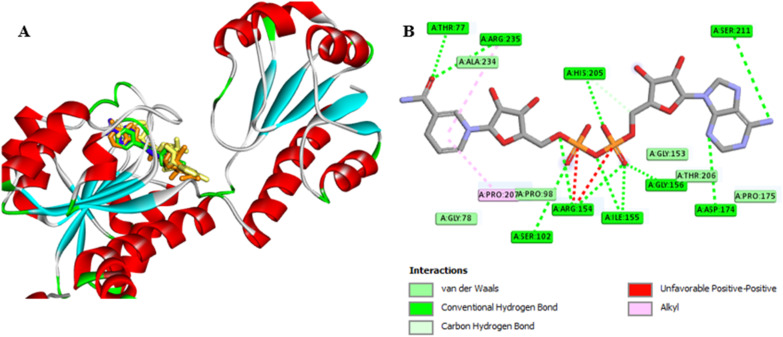
(A) 3D conformations of the native (orange), re-docked ligand (yellow), compound 7a (green), and compound 7g (blue) within the active site of human 3-phosphoglycerate dehydrogenase (PDB ID: 2G76) indicating that they are superimposed in the same position. (B) 2D conformations of the re-docked co-crystalline ligand NAD within the active site of human 3-phosphoglycerate dehydrogenase (PDB ID: 2G76).

**Fig. 10:**
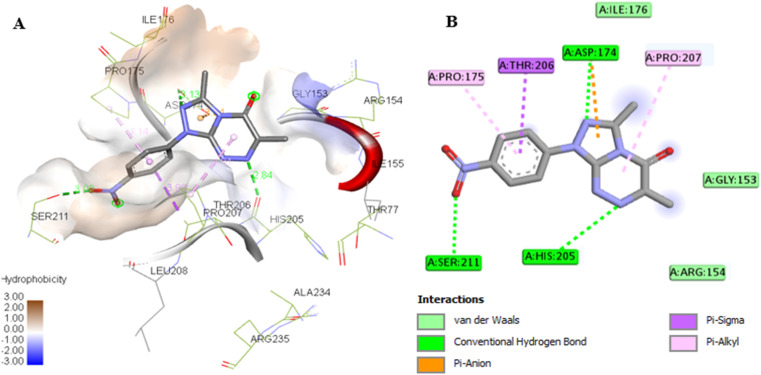
(A) 3D conformations of compound 7a within the active site of human 3-phosphoglycerate dehydrogenase (PDB ID: 2G76); (B) 2D conformations of compound 7a within the active site of human 3-phosphoglycerate dehydrogenase (PDB ID: 2G76).

**Fig. 11:**
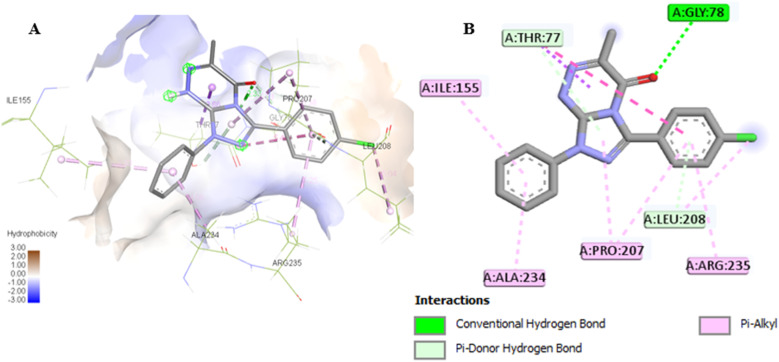
(A) 3D conformations of compound 7g within the active site of human 3-phosphoglycerate dehydrogenase (PDB ID: 2G76). (B) 2D conformations of compound 7g within the active site of human 3-phosphoglycerate dehydrogenase (PDB ID: 2G76).

**Fig. 12:**
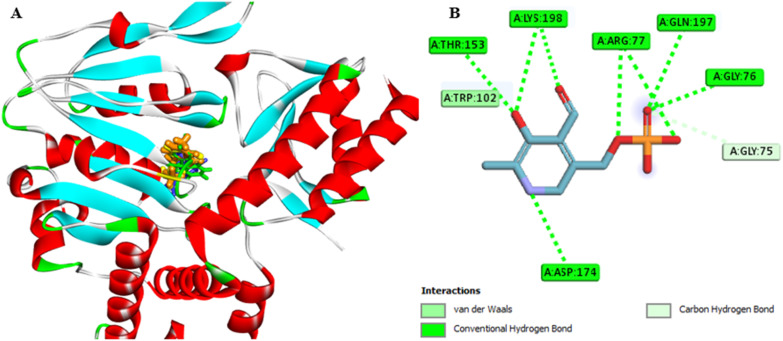
(A) 3D conformations of the native (orange), re-docked ligand (yellow), compound 7a (green), and compound 7g (blue) within the active site of phosphoserine aminotransferase (PDB ID: 7T7J) indicating that they are superimposed in the same position. (B) 2D conformations of the re-docked co-crystalline ligand PLP within the active site of phosphoserine aminotransferase (PDB ID: 7T7J).

**Fig. 13:**
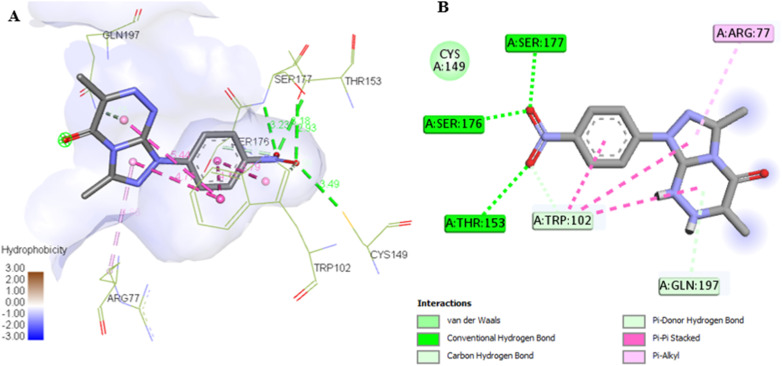
(A) 3D conformations of compound 7a within the active site of phosphoserine aminotransferase (PDB ID: 7T7J). (B) 2D conformations of compound 7a within the active site of phosphoserine aminotransferase (PDB ID: 7T7J).

**Fig. 14:**
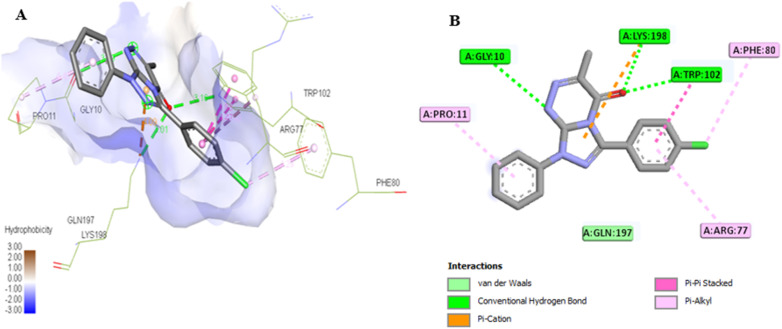
(A) 3D conformations of compound 7g within the active site of phosphoserine aminotransferase (PDB ID: 7T7J). (B) 2D conformations of compound 7g within the active site of phosphoserine aminotransferase (PDB ID: 7T7J).

Autodock Vina within PyRx software version 8 was utilized to conduct the docking simulations, and the binding energy was used to report the results for each compound. A lower binding energy value signifies a stronger binding affinity. Hydrogen bonds, the strongest interactions, were examined in conjunction with hydrophobic bonds (carbon–hydrogen, van der Waals, Pi–anion, Pi–cation, Pi–sigma, alkyl, Pi–alkyl, *etc.*). The binding affinity and interaction characteristics of the selected compounds with the target enzymes are depicted in Table 5. The 2D and 3D docked poses, along with the interactions of the co-crystallized ligand, are illustrated in Fig. 9–14. The grid box with the *XYZ* dimensions 10.19 Å × 29.38 Å × −2.48 Å and center 25.05, 19.72, 23.93 (*XYZ* coordinates) was defined to cover the 3-phosphoglycerate dehydrogenase binding while for the phosphoserine aminotransferase enzyme (PDB ID: 7T7J), the grid box was identified to cover pyridoxal-5′-phosphate (PLP) with dimensions 16.00 Å × 14.48 Å × 18.09 Å and a center 14.85, 5.89, 11.21 (*X*, *Y*, *Z*).

### Assessment of the binding affinity of compounds 7a and 7g within the active site of human 3-phosphoglycerate dehydrogenase (PDB ID: 2G76)

Firstly, the docking setup was validated by the self-docking of the co-crystallized ligand NAD into the PHGDH active pocket (PDB ID: 2G76). The docking result indicates that the re-docked NAD was superimposed on the native ligand with a docking score of −7.7 kcal mol^−1^ and formed multiple interactions with the PHGDH active site (Fig. 9A and B). The NAD revealed intermolecular hydrogen bonds with Thr77, Arg235, His205, Ser102, Arg154, Ile155, Gly156, Ser211, and Asp174 and interacted *via* alkyl and carbon–hydrogen hydrophobic interactions with the Arg235, Pro207, and His205 amino acids of the binding site (Fig. 9B). Furthermore, compounds 7a and 7g were then docked into the PHGDH active site, which demonstrated better docking scores of −7.8 and −8.6 kcal mol^−1^ than NAD, respectively, and both of them were superimposed on the same position as the co-crystalline ligand (Table 5 and Fig. 9A). Compound 7a formed a stable complex with the enzyme active pocket by establishing three hydrogen bonds with Ser211, His205, Asp174, Ile176, Gly153, and Arg154 residues, along with various hydrophobic π–alkyl, π–sigma, π–anion, and van der Waals interactions similar to the co-crystalline ligand (Fig. 10). On the other hand, compound 7g exhibited one hydrogen bond with Gly78, in addition to the π-donor H-bond, π–alkyl with Thr77, Ile155, Ala234, Pro207, and Arg235 amino acids (Fig. 11).

### Assessment of the binding affinity of compounds 7a and 7g within the active site of phosphoserine aminotransferase (PDB ID: 7T7J)

The co-crystallized ligand, pyridoxal-5′-phosphate (PLP), was re-docked into the active site of PSAT1, and the results showed that the re-docked PLP superimposed on the native ligand, achieving a docking score of −6.5 kcal mol^−1^ (Table 5 and Fig. 9A and B). Analysis of the binding site revealed that PLP formed hydrogen bonds, a carbon H-bond, and van der Waals interactions with the amino acids Thr153, Lys198, Arg77, Gln197, Gly75, Asp174, and Trp102 (Table 5 and Fig. 12B).

The docking results for compounds 7a and 7g revealed docking scores of −7.4 and −8.5, respectively, outperforming the co-crystalline ligand PLP. Both compounds formed stable complexes with the PSAT1 active site and aligned with the same position as PLP (Table 5 and Fig. 12A). Compound 7a established four hydrogen bonds with Ser177, Ser176, and Thr153, along with hydrophobic interactions involving Trp102, Gln197, Arg77, and Cys149 residues (Fig. 13). On the other hand, compound 7g displayed three hydrogen bonds with Lys198, Trp102, and Gly10, in addition to the six pi–pi stacked, pi–alkyl, pi–cation, and van der Waals interactions with the amino acids Pro11, Phe80, Trp102, Arg77, Lys198, and Gln197 similar to the co-crystalline ligand (Fig. 14).

## Materials and methods

### Chemistry

Melting points were measured with an Electrothermal 9100 apparatus and were uncorrected. The IR spectra were recorded using a FTIR Bruker-vector 22 spectrophotometer and KBr pellets. The ^1^H and ^13^C NMR spectra were recorded in CDCl_3_ or DMSO-*d*_6_ as a solvent on the Varian Gemini NMR spectrometer at 300 MHz and 75 MHz, respectively. Chemical shifts were reported as *δ* values in ppm. The elemental analyses were performed at the Microanalytical Center, Cairo University. 6-Methyl-3-thioxo-3,4-dihydro-1,2,4-triazin-5(2*H*)-one 6 ^16^ and hydrazonoyl halides 1 ^17–21^ were prepared using the reported procedures.

#### Synthesis of 6-methyl-[1,2,4]triazolo[4,3-*b*][1,2,4]triazin-7(1*H*)-one derivatives (7a–j)

To a mixture of hydrazonoyl halides 1a–j (2 mmol) and 6-methyl-3-thioxo-3,4-dihydro-1,2,4-triazin-5(2*H*)-one 3 (0.29 g, 2 mmol) in chloroform (20 mL), triethylamine (0.2 mL, 2 mmol) was added. The reaction mixture was refluxed for 6 h and then cooled; the excess chloroform was removed under reduced pressure and the residue was treated with ethanol (10 mL). The precipitated solid was collected and crystallized from a suitable solvent to give [1,2,4]triazolo[4,3-*b*][1,2,4]triazin-7-one derivatives (7a–j). All the synthesized compounds and their physical properties are listed below:

##### 3,6-Dimethyl-1-(4-nitrophenyl)-[1,2,4]triazolo[4,3-*b*][1,2,4]triazin-7(1*H*)-one (7a)

Brown crystals (EtOH + DMF); mp 262–264 °C; yield (67%); ^1^H NMR (300 MHz, DMSO-*d*_6_) *δ* 2.32 (s, 3H, CH_3_), 2.58 (s, 3H, CH_3_), 8.38 (d, 2H, 4-NO_2_C_6_H_4_, *J* ≈ 9 Hz), 8.47 (d, 2H, 4-NO_2_C_6_H_4_, *J* ≈ 9 Hz); ^13^C NMR (75 MHz, DMSO-*d*_6_) *δ* 16.5, 18.4, 118.9, 124.5, 138.9, 145.7, 151.8, 153.0, 155.3, 161.7. Anal. calcd for C_12_H_10_N_6_O_3_ (286.25), C, 50.35; H, 3.52; N, 29.36. Found, C, 50.46; H, 3.39; N, 29.47.

##### 3-Ethyl-6-methyl-1-(4-nitrophenyl)-[1,2,4]triazolo[4,3-*b*][1,2,4]triazin-7(1*H*)-one (7b)

Brown crystals (EtOH); mp 200–202 °C; yield (69%). ^1^H NMR (300 MHz, CDCl_3_) *δ* 1.48 (t, 3H, CH_2_CH̲_3_, *J* ≈ 8 Hz), 2.45 (s, 3H, CH_3_), 3.03 (q, 2H, CH̲_2_CH_3_, *J* ≈ 8 Hz), 8.30 (d, 2H, 4-NO_2_C_6_H_4_, *J* ≈ 9 Hz), 8.45 (d, 2H, 4-NO_2_C_6_H_4_, *J* ≈ 9 Hz); ^13^C NMR (75 MHz, CDCl_3_) *δ* 9.7, 17.5, 18.2, 119.5, 124.7, 141.0, 145.2, 147.9, 148.0, 155.7, 161.5. Anal. calcd for C_13_H_12_N_6_O_3_ (300.28), C, 52.00; H, 4.03; N, 27.99. Found, C, 52.11; H, 4.15; N, 27.86.

##### 6-Methyl-1-(4-nitrophenyl)-3-propyl-[1,2,4]triazolo[4,3-*b*][1,2,4]triazin-7(1*H*)-one (7c)

Brown crystals (DMF); mp 202–204 °C; yield (64%). ^1^H NMR (300 MHz, CDCl_3_) *δ* 1.13 (t, 3H, CH_2_CH_2_CH̲_3_, *J* ≈ 8 Hz), 1.91–1.99 (m, 2H, CH_2_CH̲_2_CH_3_), 2.48 (s, 3H, CH_3_), 3.00 (t, 2H, CH̲_2_CH_2_CH_3_, *J* ≈ 8 Hz), 8.34 (d, 2H, 4-NO_2_C_6_H_4_, *J* ≈ 9 Hz), 8.49 (d, 2H, 4-NO_2_C_6_H_4_, *J* ≈ 9 Hz); ^13^C NMR (75 MHz, CDCl_3_) *δ* 13.6, 18.4, 19.2, 25.6, 119.7, 124.9, 141.2, 145.5, 147.0, 148.1, 156.0, 161.6. Anal. calcd for C_14_H_14_N_6_O_3_ (314.31), C, 53.50; H, 4.49; N, 26.74. Found, C, 53.61; H, 4.37; N, 26.87.

##### 3-Isopropyl-6-methyl-1-(4-nitrophenyl)-[1,2,4]triazolo[4,3-*b*][1,2,4]triazin-7(1*H*)-one (7d)

Brown crystals (EtOH); mp 208–210 °C; yield (66%). ^1^H NMR (300 MHz, CDCl_3_) *δ* 1.52 (d, 6H, CH(CH̲_3_)_2_, *J* ≈ 7 Hz), 2.47 (s, 3H, CH_3_), 3.43–3.52 (m, 1H, CH̲(CH_3_)_2_), 8.32 (d, 2H, 4-NO_2_C_6_H_4_, *J* ≈ 9 Hz), 8.48 (d, 2H, 4-NO_2_C_6_H_4_, *J* ≈ 9 Hz); ^13^C NMR (75 MHz, CDCl_3_) *δ* 18.3, 19.1, 24.9, 119.6, 124.7, 141.1, 145.2, 148.1, 150.9, 155.6, 161.5. Anal. calcd for C_14_H_14_N_6_O_3_ (314.31), C, 53.50; H, 4.49; N, 26.74. Found, C, 53.60; H, 4.38; N, 26.86.

##### 3-Isobutyl-6-methyl-1-(4-nitrophenyl)-[1,2,4]triazolo[4,3-*b*][1,2,4]triazin-7(1*H*)-one (7e)

Brown crystals (EtOH); mp 202–204 °C; yield (63%). ^1^H NMR (300 MHz, CDCl_3_) *δ* 1.12 (d, 6H, CH_2_CH(CH̲_3_)_2_, *J* ≈ 7 Hz), 2.30–2.39 (m, 1H, CH_2_CH̲(CH_3_)_2_), 2.48 (s, 3H, CH_3_), 2.93 (d, 2H, CH̲_2_CH(CH_3_)_2_, *J* ≈ 7 Hz), 8.36 (d, 2H, 4-NO_2_C_6_H_4_, *J* ≈ 9 Hz), 8.50 (d, 2H, 4-NO_2_C_6_H_4_, *J* ≈ 9 Hz); ^13^C NMR (75 MHz, CDCl_3_) *δ* 18.4, 22.3, 26.4, 32.2, 94.7, 119.7, 124.9, 141.1, 145.5, 146.4, 156.0, 161.6. Anal. calcd for C_15_H_16_N_6_O_3_ (328.33), C, 54.87; H, 4.91; N, 25.60. Found, C, 54.99; H, 4.79; N, 25.72.

##### 6-Methyl-1-phenyl-3-(*p*-tolyl)-[1,2,4]triazolo[4,3-*b*][1,2,4]triazin-7(1*H*)-one (7f)

Beige crystals (DMF); mp 250–252 °C; yield (72%). ^1^H NMR (300 MHz, CDCl_3_) *δ* 2.47 (s, 3H, CH_3_), 2.53 (s, 3H, CH_3_), 7.33–7.38 (m, 3H, Ar-H), 7.51 (t, 2H, Ar-H, *J* ≈ 8 Hz), 8.18–8.24 (m, 4H, Ar-H); ^13^C NMR (75 MHz, CDCl_3_) *δ* 18.4, 20.9, 121.6, 125.1, 126.4, 128.7, 129.2, 131.5, 138.5, 140.9, 147.9, 151.4, 152.8, 161.7. Anal. calcd for C_18_H_15_N_5_O (317.35), C, 68.13; H, 4.76; N, 22.07. Found, C, 68.25; H, 4.63; N, 22.18.

##### 3-(4-Methoxyphenyl)-6-methyl-1-phenyl-[1,2,4]triazolo[4,3-*b*][1,2,4]triazin-7(1*H*)-one (7g)

White crystals (DMF); mp 256–258 °C; yield (70%). ^1^H NMR (300 MHz, DMSO-*d*_6_) *δ* 2.34 (s, 3H, CH_3_), 3.87 (s, 3H, OCH_3_), 7.20 (d, 2H, Ar-H, *J* ≈ 9 Hz), 7.42 (t, 1H, Ar-H, *J* ≈ 8 Hz), 7.60 (t, 2H, Ar-H, *J* ≈ 8 Hz), 8.12 (d, 2H, Ar-H, *J* ≈ 9 Hz), 8.19 (d, 2H, Ar-H, *J* ≈ 9 Hz); ^13^C NMR (75 MHz, DMSO-*d*_6_) *δ* 18.2, 55.5, 113.8, 120.7, 122.8, 126.3, 128.9, 130.8, 138.4, 147.9, 151.4, 152.6, 160.4, 161.8. Anal. calcd for C_18_H_15_N_5_O_2_ (333.35), C, 64.86; H, 4.54; N, 21.01. Found, C, 64.98; H, 4.41; N, 21.14.

##### 3-(4-Chlorophenyl)-6-methyl-1-phenyl-[1,2,4]triazolo[4,3-*b*][1,2,4]triazin-7(1*H*)-one (7h)

Yellow crystals (DMF); mp 245–247 °C; yield (74%). ^1^H NMR (300 MHz, CDCl_3_) *δ* 2.54 (s, 3H, CH_3_), 7.38 (t, 1H, Ar-H, *J* ≈ 8 Hz), 7.49–7.58 (m, 4H, Ar-H), 8.22 (d, 2H, Ar-H, *J* ≈ 8 Hz), 8.31 (d, 2H, Ar-H, *J* ≈ 9 Hz); ^13^C NMR (75 MHz, CDCl_3_) *δ* 18.6, 120.4, 122.0, 127.5, 128.0, 129.3, 131.9, 136.0, 138.1, 148.0, 151.9, 155.9, 161.9. Anal. calcd for C_17_H_12_ClN_5_O (337.77), C, 60.45; H, 3.58; N, 20.73. Found, C, 60.57; H, 3.72; N, 20.86.

##### 3-(Furan-2-yl)-6-methyl-1-(4-nitrophenyl)-[1,2,4]triazolo[4,3-*b*][1,2,4]triazin-7(1*H*)-one (7i)

Pale brown crystals (CH_3_CN); mp 269–271 °C; yield (71%). ^1^H NMR (300 MHz, DMSO-*d*_6_) *δ* 2.39 (s, 3H, CH_3_), 6.87–6.89 (m, 1H, furyl-H), 7.66 (d, 1H, furyl-H, *J* ≈ 3 Hz), 8.15 (d, 1H, furyl-H, *J* ≈ 1 Hz), 8.41–8.49 (m, 4H, 4-NO_2_C_6_H_4_); ^13^C NMR (75 MHz, DMSO-*d*_6_) *δ* 18.2, 112.6, 117.1, 119.8, 125.3, 136.5, 137.4, 141.2, 145.0, 147.1, 148.3, 154.9, 160.8. Anal. calcd for C_15_H_10_N_6_O_4_ (338.28), C, 53.26; H, 2.98; N, 24.84. Found, C, 53.38; H, 2.86; N, 24.95.

##### 6-Methyl-1-(4-nitrophenyl)-3-(thiophen-2-yl)-[1,2,4]triazolo[4,3-*b*][1,2,4]triazin-7(1*H*)-one (7j)

Pale brown crystals (DMF); mp 242–244 °C; yield (69%). ^1^H NMR (300 MHz, DMSO-*d*_6_) *δ* 2.38 (s, 3H, CH_3_), 7.36 (t, 1H, thieny-H, *J* ≈ 5 Hz), 8.05 (d, 1H, thieny-H, *J* ≈ 5 Hz), 8.22 (d, 1H, thieny-H, *J* ≈ 5 Hz), 8.38–8.47 (m, 4H, 4-NO_2_C_6_H_4_); ^13^C NMR (75 MHz, DMSO-*d*_6_) *δ* 18.3, 119.7, 120.4, 123.4, 125.3, 128.6, 131.7, 132.2, 139.9, 141.1, 144.9, 154.8, 160.8. Anal. calcd for C_15_H_10_N_6_O_3_S (354.34), C, 50.84; H, 2.84; N, 23.72. Found, C, 50.95; H, 2.71; N, 23.85.

### Anti-cancer activity

#### MTT cytotoxicity assay

Cell viability was tested using the MTT assay, 3-(4,5-dimethylthiazol-2-yl)-2,5-diphenyl-tetrazolium bromide (Bio Basic Canada Inc. Toronto, Canada).^26^ Under class II biosafety regulations, the procedures were carried out in a sterile laminar air flow cabinet (Baker, SG403INT; Sanford, ME, USA). Cell lines were obtained from the American type culture collection (ATCC) as a gift from Dr Stig Linder, Karonisca Institute, Sweden. All incubations were performed at 37 °C in a 5% CO_2_ incubator with a 95% humidified environment (Sheldon, TC2323; Cornelius, OR, USA). 96-well micro titer polypropylene plates were seeded with cells at a density of 10^4^ cells per well in complete DMEM media, and the cells were allowed to adhere for 24 hours. After the media was aspirated, the tested compounds were added to the cells in a single dose of 100 μg ml^−1^ in DMSO. Each well received 40 μl of MTT salt (2.5 g ml^−1^) following a 48 hour incubation period. 200 μl of 10% sodium dodecyl sulfate (SDS) was added to each well after the reaction ended, and any formazan crystals that could have been produced were dissolved by two hours of incubator heating to 37 °C. The amount of formazan product was measured at 595 nm using a microplate reader (Bio-Rad Laboratories, model 3350, California, USA) with a reference wavelength of 690 nm, serving as a backdrop. Rather than the drugs under investigation, the medium was applied to the untreated cells (negative control). The examined compounds were dissolved in dimethylsulfoxide (DMSO), and the final concentration in the cells was less than 0.2%. For each compound and the control, the solvent concentration was the same. By applying different concentrations of 0, 6.25, 12.2, 25, and 50 μg ml^−1^ (three replicates), the concentration required for 50% inhibition of cell viability (IC_50_) was estimated for the potential compounds, which demonstrated preliminary cytotoxic effects at 100 μg ml^−1^.

### Gene expression analysis

#### Quantitative real-time PCR method

##### RNA isolation and reverse transcription (RT) reaction

The RNeasy Mini Kit (Qiagen, Hilden, Germany), augmented with a DNaseI (Qiagen) digestion step, was employed to extract total RNA from lung cancer cell lines following the manufacturer's procedure. The isolated total RNA was treated with one unit of RQ1 RNAse-free DNAse (Invitrogen, Germany) to eliminate DNA remnants, re-suspended in DEPC-treated water, and quantified photometrically at 260 nm. The purity of total RNA was evaluated using the 260/280 nm ratio, which ranged from 1.8 to 2.1.^27^

Furthermore, integrity was confirmed using ethidium bromide-stained examination of 28S and 18S bands *via* formaldehyde-containing agarose gel electrophoresis. Aliquots were utilized immediately for reverse transcription (RT); otherwise, they were preserved at −80 °C.

The poly(A) + RNA extracted from the lung cancer cell lines was reverse transcribed into the cDNA in a total amount of 20 μl utilizing the Revert Aid TM First Strand cDNA Synthesis Kit (Fermentas, Germany). A total of 5 μg of RNA was utilized with a master mix. The master mix comprised 50 mM MgCl_2_, 10× RT buffer (50 mM KCl; 10 mM Tris–HCl; pH 8.3), 10 mM of each dNTP, 50 μM oligo-dT primer, 20 IU ribonuclease inhibitor (50 kDa recombinant enzyme to suppress RNase activity), and 50 IU MuLV reverse transcriptase. Each sample mixture was centrifuged for 30 seconds at 1000 g and thereafter transferred to the thermocycler. The RT reaction was conducted at 25 °C for 10 minutes, subsequently at 42 °C for 1 hour, and concluded with a denaturation phase at 99 °C for 5 minutes. Subsequently, the reaction tubes containing RT-prepared samples were rapidly chilled in an ice chamber prior to cDNA amplification *via* the quantitative real-time polymerase chain reaction (qRT-PCR).

##### Quantitative real-time-PCR (qRT-PCR)

The cDNA copy number of lung cancer cell lines was determined using the StepOne™ Real-Time PCR System from Applied Biosystems (Thermo Fisher Scientific, Waltham, MA, USA).

The PCR reactions were prepared in 25 μl mixes comprising 12.5 μl of 1× SYBR® Premix Ex Taq™ (TaKaRa, Biotech. Co. Ltd), 0.5 μl of 0.2 μM sense primer, 0.5 μl of 0.2 μM antisense primer, 6.5 μl of distilled water, and 5 μl of cDNA template.

The reaction protocol was divided into three stages. The initial step was conducted at 95 °C for a duration of 3 minutes. The second step comprised 40 cycles, each divided into three phases: (a) 95 °C for 15 seconds, (b) 55 °C for 30 seconds, and (c) 72 °C for 30 seconds. The third stage comprised 71 cycles, first at 60 °C and subsequently increasing by approximately 0.5 °C every 10 seconds until 95 °C. Every experiment incorporated a distilled water control. The particular primer sequences for the lung cancer cell lines (BCL-2, BAX, and p53 genes, Brito *et al.*, 2012)^28^ were created and are presented in Table 6. A melting curve analysis was conducted at 95.0 °C after each qPCR to assess the quality of the utilized primers. The relative quantification of the target gene to the reference gene was assessed using the 2^−ΔΔ*C*_T_^ method.^29–31^

**Table 6:** Primer sequences used for the qRT-PCR of lung cancer cell lines^a^

Gene	Primer sequence	GenBank (accession no.)
BCL-2	F: TTCCGCGTGATTGAAGACAC	KY098818.1
	R: ACTTCATCACTATCTCCCGGT	
p53	F: GGAAATCTCACCCCATCCCA	AB082923.1
	R: CAGTAAGCCAAGATCACGCC	
BAX	F: AACATGGAGCTGCAGAGGAT	L22474.1
	R: CCAATGTCCAGCCCATGATG	
GAPDH	F: AGGTCGGAGTCAACGGATTT	NM_001357943.2
	R:ATCGCCCCACTTGATTTTGG	

a BCL-2: B-cell lymphoma-2 gene; BAX: BCL-2-associated X protein-encoding gene; p53: tumor suppressor gene; GAPDH: glyceraldehyde-3-phosphate dehydrogenase.

### DNA damage evaluation using the comet assay

The determination of DNA damage *via* the comet assay was conducted using lung cancer cell lines, following the methodology established by Olive *et al.* (1990).^32^ Following trypsin treatment to generate a single-cell suspension, approximately 1.5 × 10^4^ cells were embedded in 0.75% low-gelling-temperature agarose and swiftly pipetted onto a pre-coated microscope slide.

The samples underwent lysis for 4 hours at 50 °C in a solution containing 0.5% SDS and 30 mM EDTA at pH 8.0. Following an overnight rinse at room temperature in a Tris/borate/EDTA buffer, pH 8.0, samples underwent electrophoresis for 25 minutes at 0.6 V cm^−1^ and were subsequently stained with propidium iodide. The slides were examined with a fluorescence microscope equipped with a CCD camera, and 150 individual comet images were analyzed from each sample for a tail moment, DNA content, and percentage of DNA in the tail. Approximately 100 cells were analyzed per sample to assess the percentage of cells exhibiting comet-like DNA damage.

The non-overlapping cells were randomly selected and assigned a score on a scale of 0–3 based on the comet tail length migration and the relative proportion of DNA in the nucleus. Class 0 indicates no detectable DNA damage and no tail; class 1 indicates a tail length less than the diameter of the nucleus; class 2 indicates a tail length between 1× and 2× the nuclear diameter; and class 3 indicates a tail longer than 2× the diameter of the nucleus (Collins *et al.*, 1997).^33^

### DNA fragmentation assay

#### DNA gel electrophoresis laddering assay

The DNA fragmentation assay in lung cancer cell lines was performed in accordance with the protocol established by Yawata (1998)^34^ with some modifications. Briefly, the lung cancer cell lines (A549) exposed to different compounds such as PT1, PT7 and Doxorubicin were homogenized in 1 ml of the medium and centrifuged (10 min at 800 rpm). The low-molecular-weight genomic DNA was extracted as described in Yawata (1998).^34^ Approximately 1 × 10^6^ cells of each treatment were plated. All the cells (including floating cells) were harvested and washed with Dulbecco's phosphate buffered saline. The cancer cells were lysed with the lysis buffer containing 10 mM Tris (pH 7.4), 150 mM NaCl, 5 mM ethylenediaminetetraacetic acid (EDTA), and 0.5% Triton X-100 for 30 min on ice. Lysates were vortexed and cleared by centrifugation at 10 000 g for 20 min. Fragmented DNA in the supernatant was extracted with an equal volume of a neutral phenol : chloroform : isoamyl alcohol mixture (25 : 24 : 1) and analyzed electrophoretically on 2% agarose gels containing 0.1 μg ml^−1^ ethidium bromide.

### Diphenylamine reaction procedure

The lung cancer cell lines (A549) were used to determine the quantitative profile of the DNA fragmentation. The cell lines were collected immediately after the culture and treatment with PT1 and PT7 as well as with Dox. The cancer cells were lysed in 0.5 ml of lysis buffer containing 10 mM Tris–HCl (pH 8), 1 mM EDTA, and 0.2% Triton X-100 and centrifuged at 10 000 rpm (Eppendorf) for 20 min at 4 °C. The pellets were re-suspended in 0.5 ml of lysis buffer. To the pellets (P) and the supernatants (S), 0.5 ml of 25% tri-chloroacetic acid (TCA) was added and incubated at 4 °C for 24 h. The cells were then centrifuged for 20 min at 10 000 rpm (Eppendorf) at 4 °C and the pellets were suspended in 80 ml of 5% TCA, followed by incubation at 83 °C for 20 min. Subsequently, to each cell sample, 160 ml of diphenylamine (DPA) solution [150 mg DPA in 10 ml glacial acetic acid, 150 ml of sulfuric acid and 50 ml acetaldehyde (16 mg ml^−1^)] was added and incubated at room temperature for 24 h (Gibb *et al.*,^35^ 1997). The proportion of the fragmented DNA was calculated from the absorbance values at 600 nm wavelength using the formula:% fragmented DNA = [OD(S)/[OD(S) + OD(P)]] × 100(OD: optical density, S: supernatants, P: pellets).

### Statistical analysis

All data were analyzed using the General Linear Model (GLM) procedure of the Statistical Analysis System (SAS) (1982),^36^ followed by the Scheffé-test to assess significant differences between groups. The values are expressed as mean ± SEM. All statements of significance were based on a probability of *P* < 0.05.

### Molecular docking simulation

The molecular docking of the most active compounds 7a and 7g with two enzymes that play an important role in lung cancer progression, namely, 3-phosphoglycerate dehydrogenase (PHGDH) and phosphoserine aminotransferase (PSAT1), was performed on Autodock 4 in PyRx software version 8.^37,38^ The 3D structures of the target enzymes were obtained from the RCSB protein data bank in the PDB format using codes 2G76 (3-phosphoglycerate dehydrogenase in complex with nicotinamide–adenine–dinucleotide (NAD)) and 7T7J (phosphoserine aminotransferase in complex with pyridoxal-5′-phosphate (PLP)).^39,40^

Before the docking process, the enzymes underwent preparation, including removal of the co-crystallized ligands and water molecules, then optimization using the *QuickPrep* tool module in the MOE program. The resulting files were saved as pdb and then converted to the PDBQT format using Autodock Vina tools. Autodock tools were employed to set the size and the center of the grid box that covers the native ligands completely. The docking protocol was validated by re-docking the co-crystallized ligands into the enzyme-active sites and the RMSD was calculated using the DockRMSD server.^41^ The chemical structure of the selected compounds 7a and 7g were constructed with the *ChemDraw* ultra-10.0, saved as SDF files, then minimized by applying the MMFF94 force field and converted to pdbqt files using OpenBable tools involved in PyRx software. PyRx software presented the 8 most suitable docking poses of the ligand–protein complex after the docking was completed and subsequently ranked according to the binding energy. We selected the first docking pose, which is the most suitable pose where the ligands have the lowest binding energy, zero Å root-mean-square deviation (RMSD) and strongly interact with the protein's catalytic cavity, and visualized using BIOVIA Discovery Studio Visualizer 2021 for insights into the ligand binding position in the protein cavity.

### Crystallography

The data for compound 7b was collected at a low temperature (100 K) on a Rigaku XtaLAB Synergy dual microfocus X-ray diffractometer, using a PhotonJet-S series of microfocus X-ray source, Cu-Kα radiation (*λ* = 1.54184 Å) and equipped with an Oxford Cryosystems Cooler Device. The cell parameters of the final unit were obtained by means of a least-squares refinement. The structure was solved by Direct Methods using SHELXT 2018/2 (ref. 42) and refined by means of least-squares procedures on a *F*^2^ using the program CRYSTALS.^43^ The Atomic Scattering Factors were taken from the International Tables for X-Ray Crystallography.^44^ All hydrogen atoms were geometrically placed and refined using a riding model.

All non-hydrogen atoms were anisotropically refined, and in the last cycles of refinement, a weighting scheme was used, where weights are calculated from the following formula:*w* = 1/[2(Fo2) + (aP)2 + bP]where *P* = (Fo2 + 2Fc2)/3. Molecules were drawn with the program ORTEP32 with 30% probability displacement ellipsoids for non-hydrogen atoms.^45^

## Conclusions

In summary, we reported a novel series of [1,2,4]triazolo[4,3-*b*][1,2,4]triazin-7-one derivatives (7a–j) by the reaction of 6-methyl-3-thioxo-3,4-dihydro-1,2,4-triazin-5(2*H*)-one with the corresponding hydrazonoyl halides in chloroform. The chemical structures of these compounds were proven using spectral data, elemental analyses, and single-crystal X-ray diffraction. The anti-cancer activity of these compounds was determined against the PC3, A549, PACA2 and BJ1 using MTT assay. Two compounds, 7a and 7g, showed potent anti-cancer activities with low IC_50_ values (36.6 and 40.1 μM, respectively) compared to the reference drug with an IC_50_ value of 43.8 μM on lung cell lines and demonstrated safe mortality effect on the normal cell line (BJ1) with cytotoxicity percentages of 3.5% and 2.8%, respectively. These compounds (7a and 7g) were investigated further to determine their mechanism of action using DNA fragmentation, DNA damage and gene expression (BCL-2, BAX, and p53 genes). Molecular docking was employed to explore the binding affinity between the most active compounds (7a and 7g) and two key enzymes, PHGDH and PSAT1, which play vital roles in the progression of lung cancer.

## Data availability

The data supporting this article (copies of ^1^H, ^13^C NMR spectra and single-crystal X-ray details) have been included as part of the ESI.†

## Conflicts of interest

There are no conflicts to declare.

## Supplementary Material

RA-015-D4RA08958H-s001

RA-015-D4RA08958H-s002
