# Supplementary material for: Utility of 6-aza-2-thiothymine in the synthesis of novel [1,2,4]triazolo[4,3-b][1,2,4]triazin-7-one derivatives: synthesis, structure elucidation, molecular docking and in vitro anti-lung cancer activity
Source: RSC Adv. 2025 Feb 26;15(8):6015–31. doi: 10.1039/d4ra08958h (PMC11862990; doi:10.1039/d4ra08958h)
Supplement: RA-015-D4RA08958H-s001 [file RA-015-D4RA08958H-s001.pdf]

Supplementary Information (SI) for RSC Advances.
This journal is © The Royal Society of Chemistry 2025

Utility of 6-aza-2-thiothymine in the synthesis of novel [1,2,4]triazolo[4,3-*b*][1,2,4]triazin-7-one derivatives: Synthesis, structure elucidation, molecular docking and *in vitro* anti-lung cancer activity

Monica G. Kamel[a], Farid M. Sroor[b,*], Khaled Mahmoud[c], Heba I Shafey[d], Hamdi M. Hassaneen[a,*], Laure Vendier[e]

[a] Department of Chemistry, Faculty of Science, Cairo University, Giza, Egypt
[b] Organometallic and Organometalloid Chemistry Department, National Research Centre, 12622 Cairo, Egypt
[c] Pharmacognosy Department, National Research Centre, 12622-Dokki, Egypt
[d] Cell Biology Department, National Research Centre, 12622-Dokki, Egypt
[e] LCC-CNRS, Université de Toulouse, CNRS, UPS, Toulouse, France

Correspondence authors

Farid M Sroor, faridsroor@gmx.de, fm.sroor@nrc.sci.eg

Hamdi M. Hassaneen, hamdi_251@yahoo.com, hhassaneen@sci.cu.edu.eg

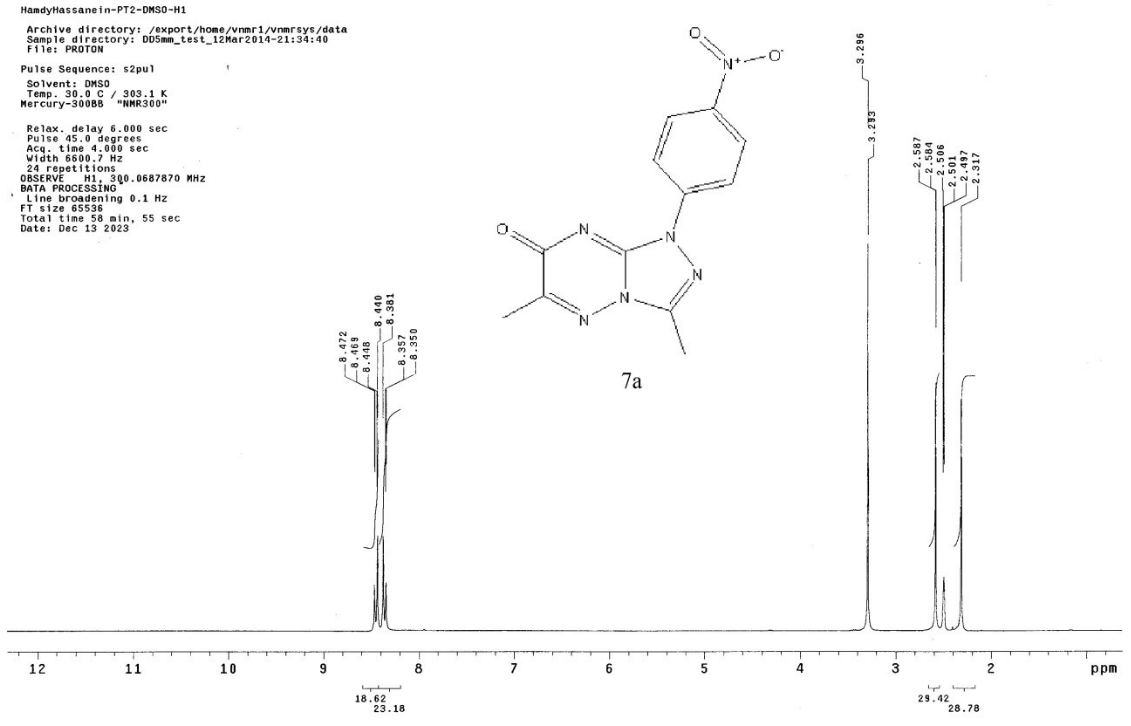

Figure S1. 1HNMR Spectrum of 7a.

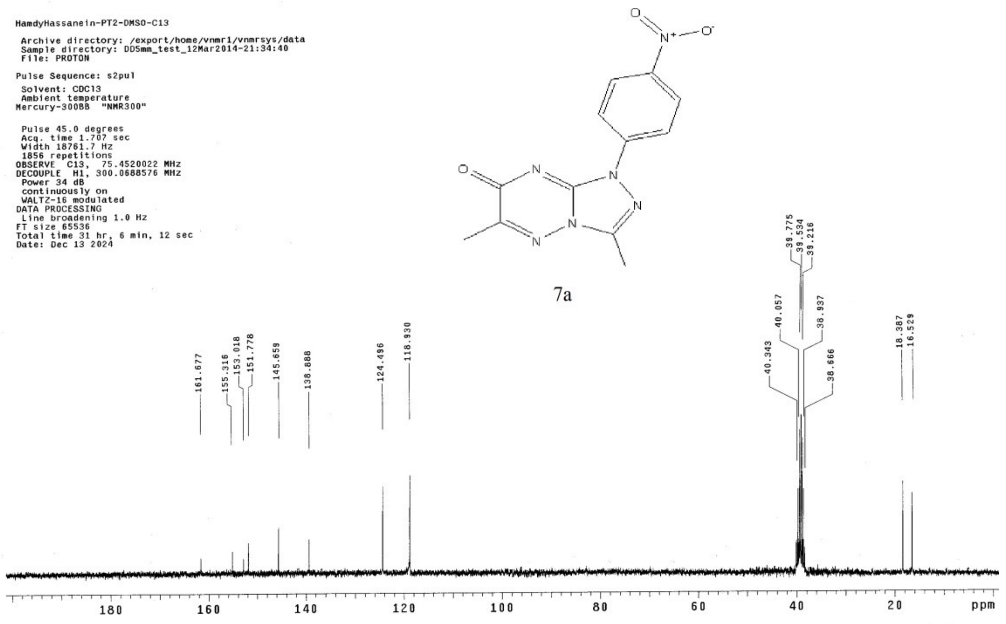

Figure S2. 13CNMR Spectrum of 7a.

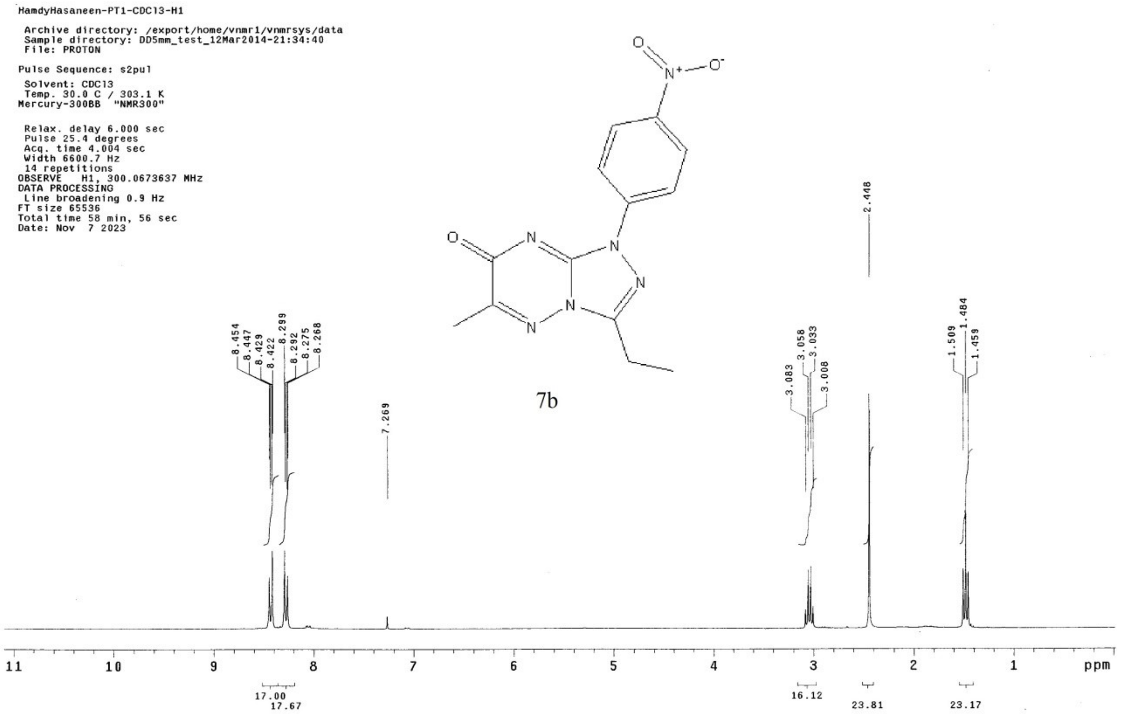

Figure S3. 1HNMR Spectrum of 7b.

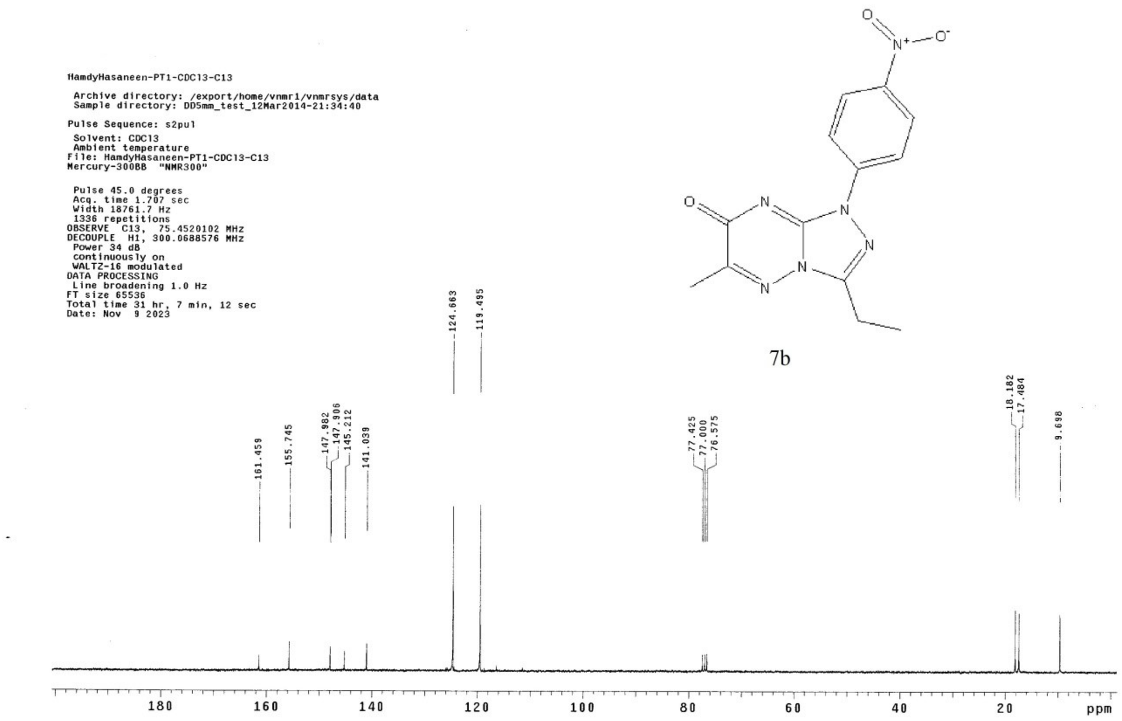

Figure S4. 13CNMR Spectrum of 7b.

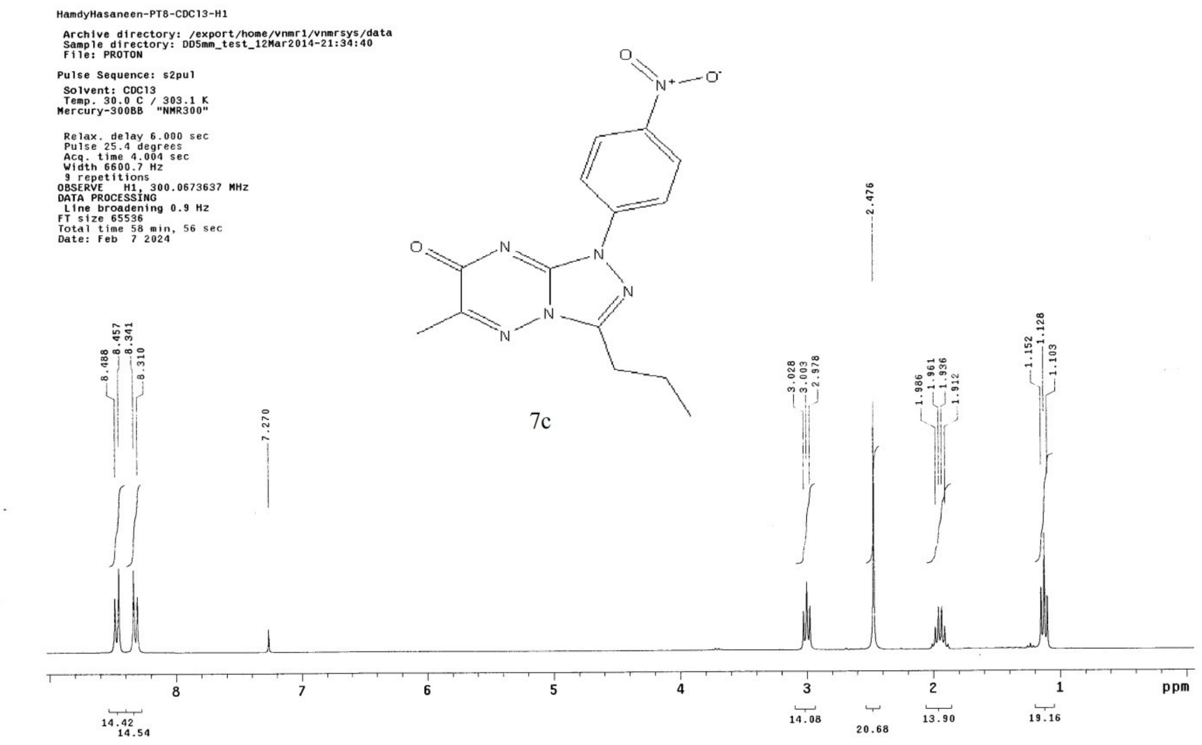

Figure S5. 1HNMR Spectrum of 7c.

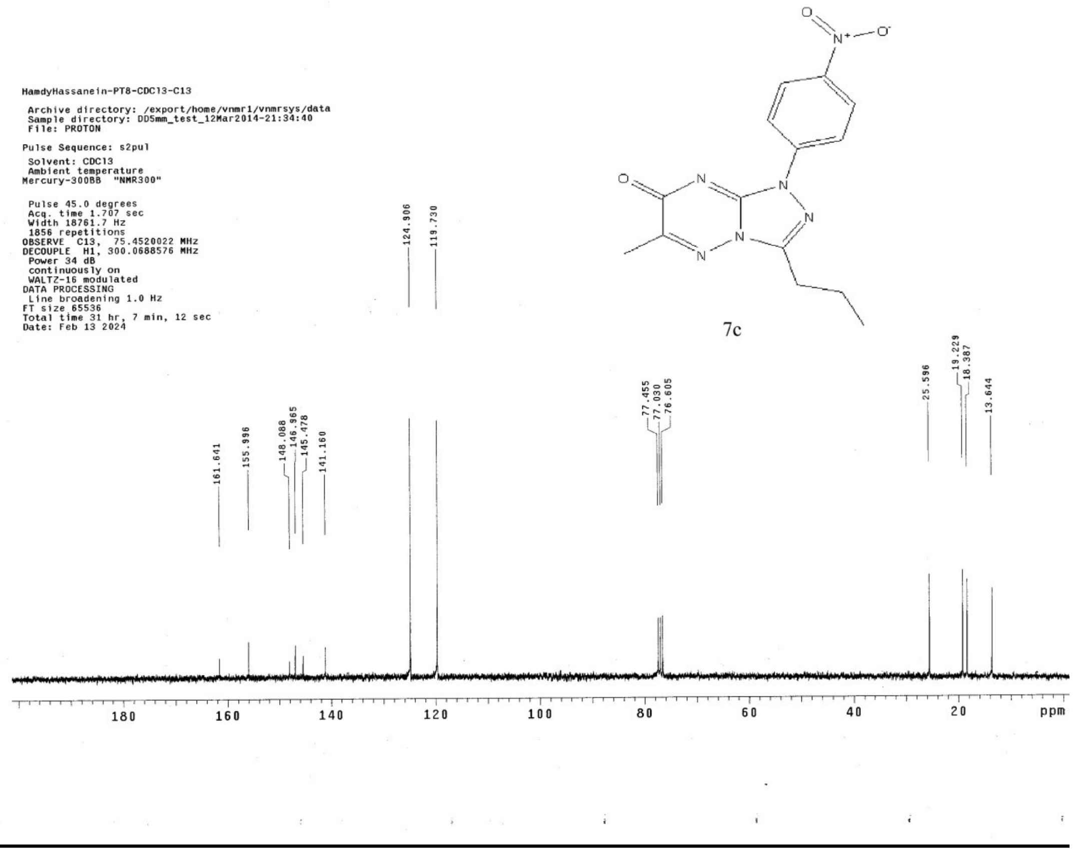

Figure S6. 13CNMR Spectrum of 7c.

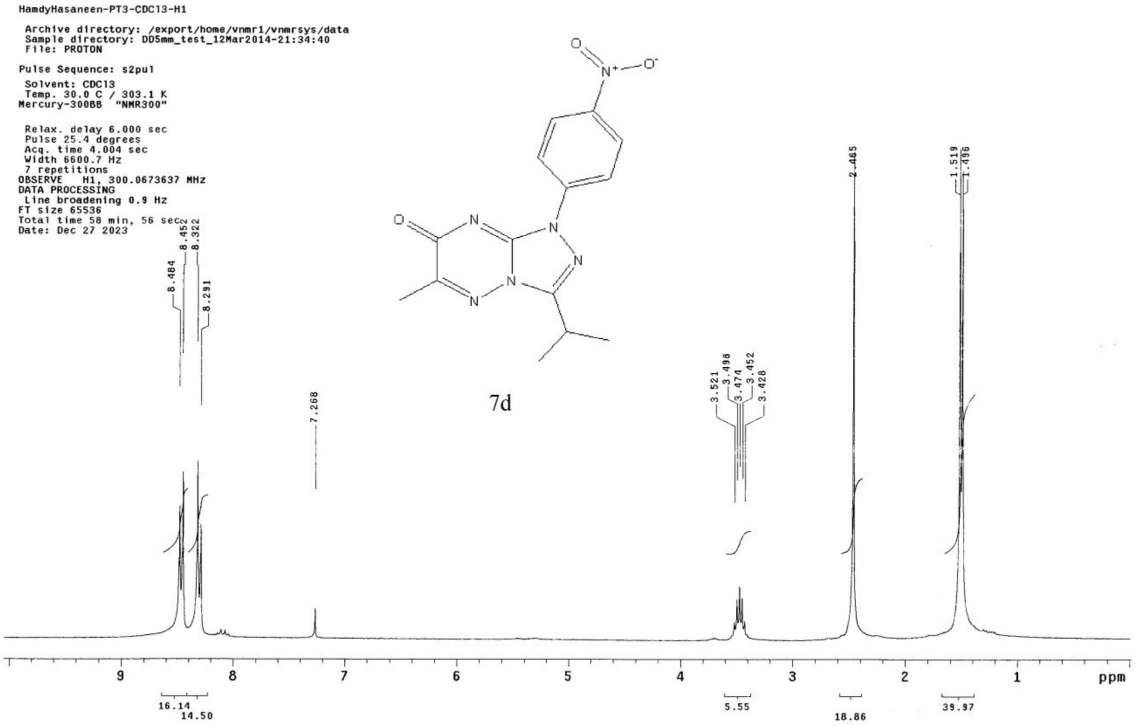

Figure S7. 1HNMR Spectrum of 7d.

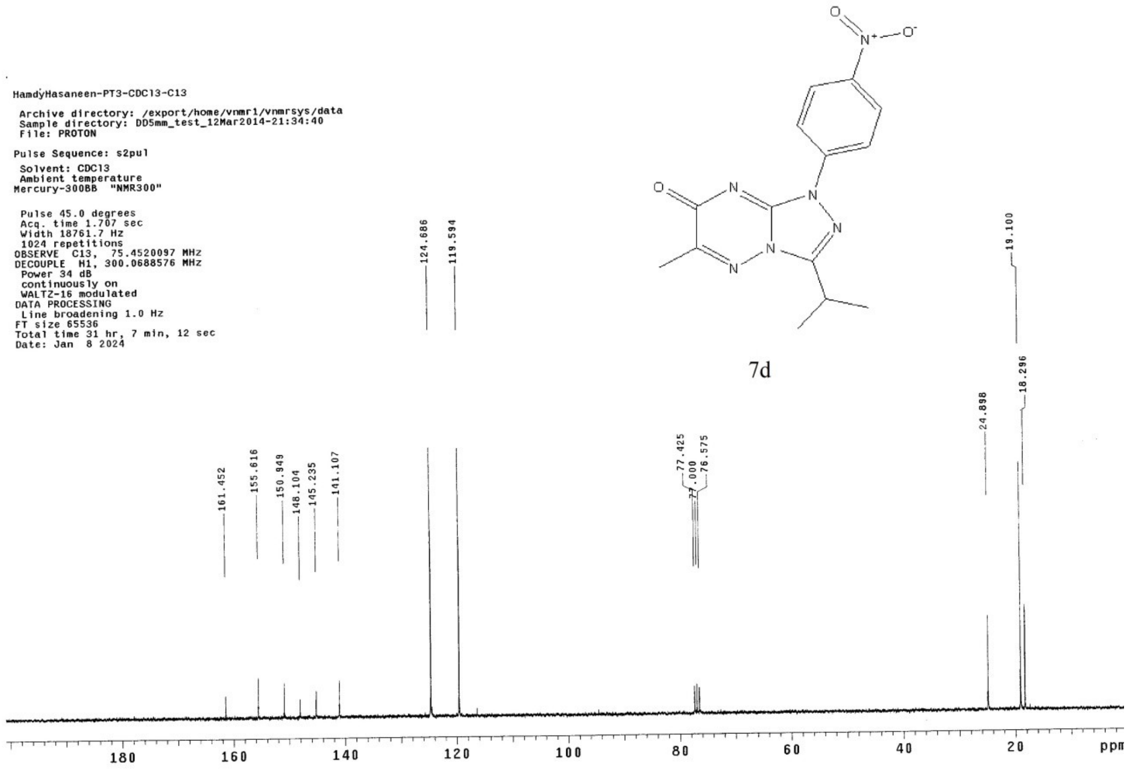

Figure S8. 13CNMR Spectrum of 7d.

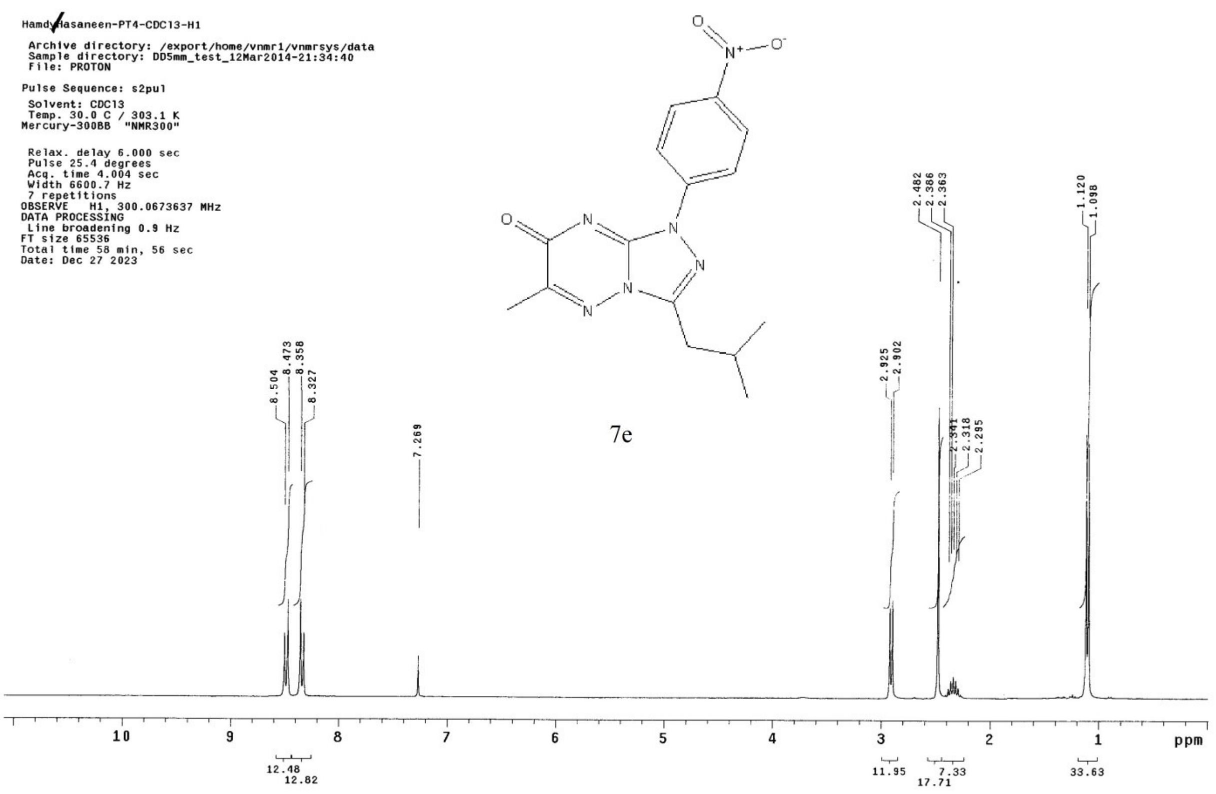

Figure S9. 1HNMR Spectrum of 7e.

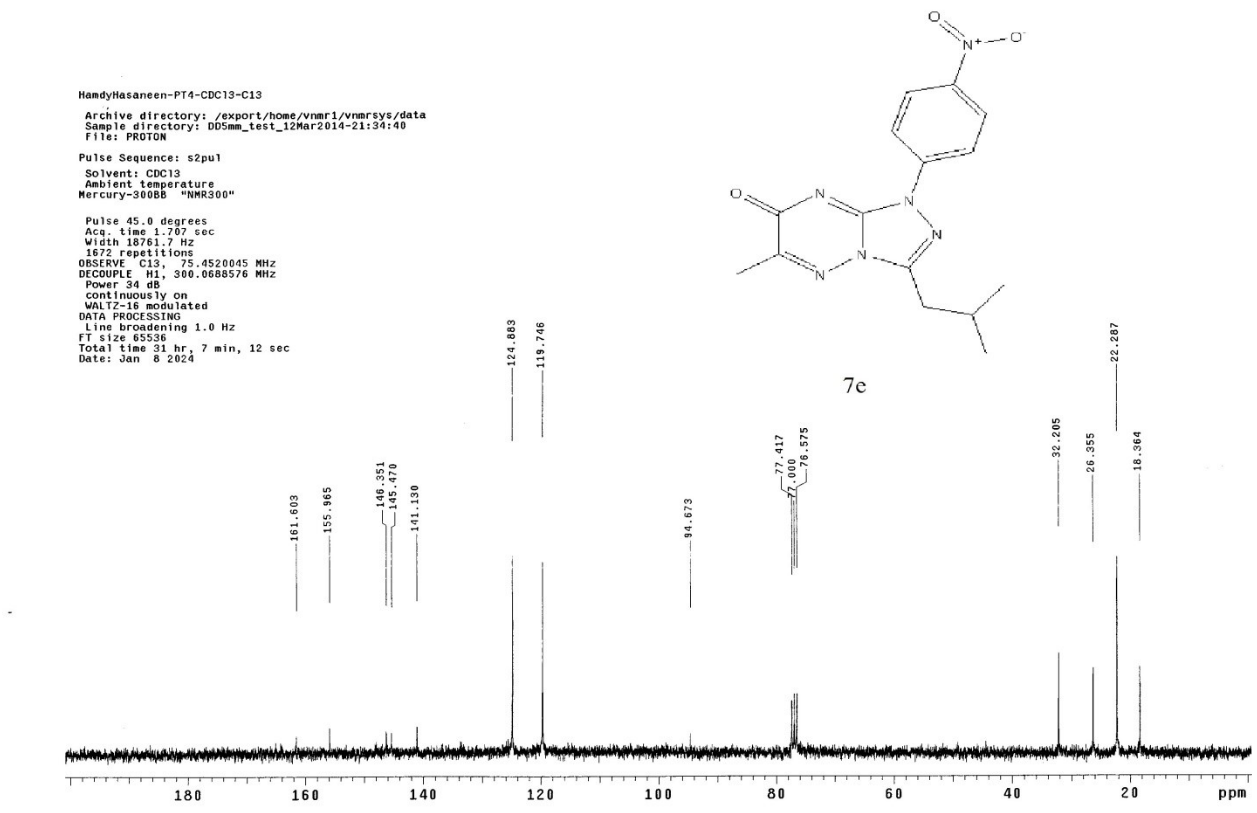

Figure S10. 13CNMR Spectrum of 7e.

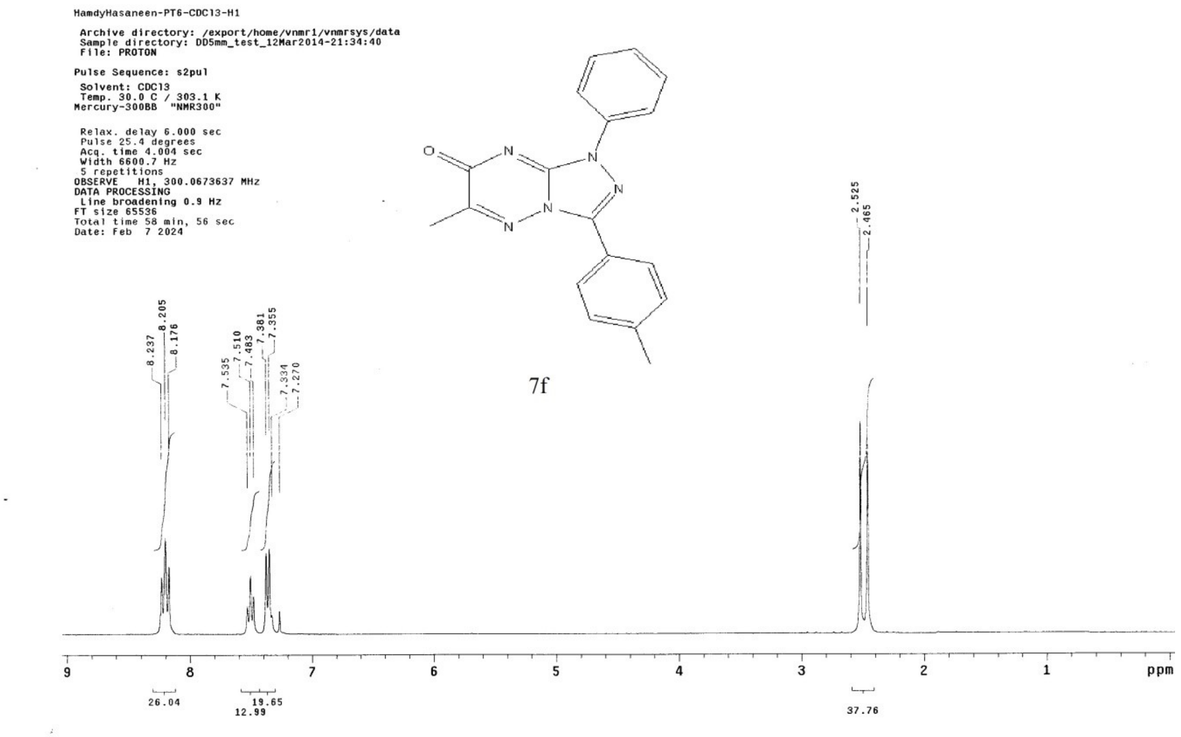

Figure S11. 1HNMR Spectrum of 7f.

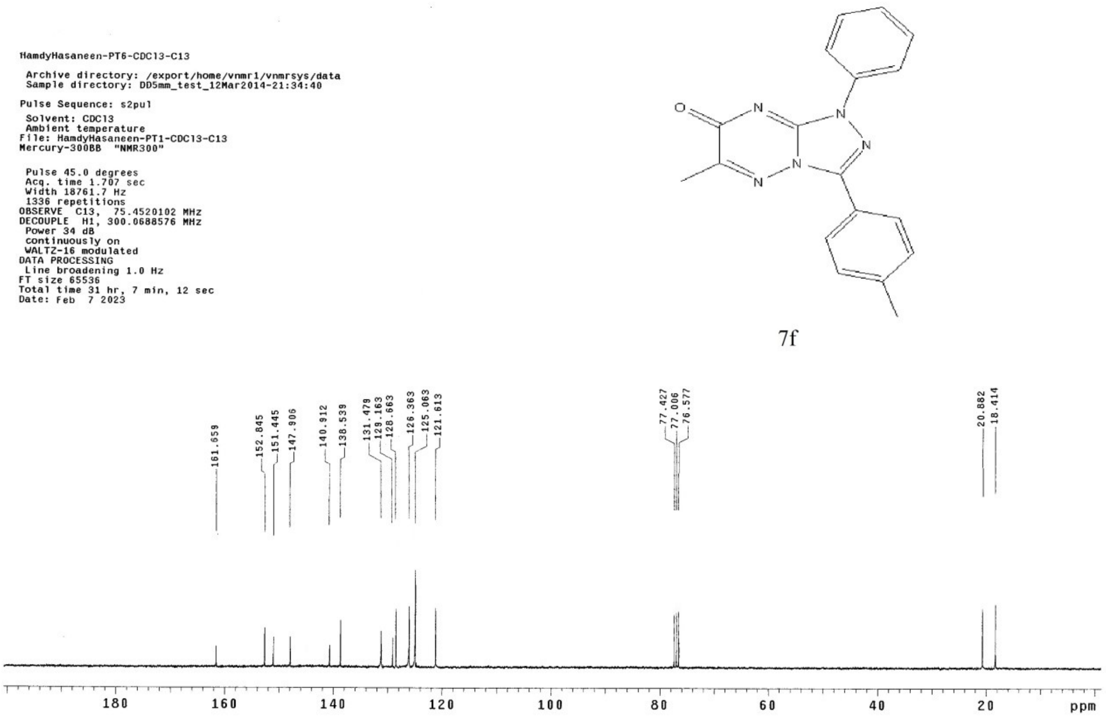

Figure S12. 13CNMR Spectrum of 7f.

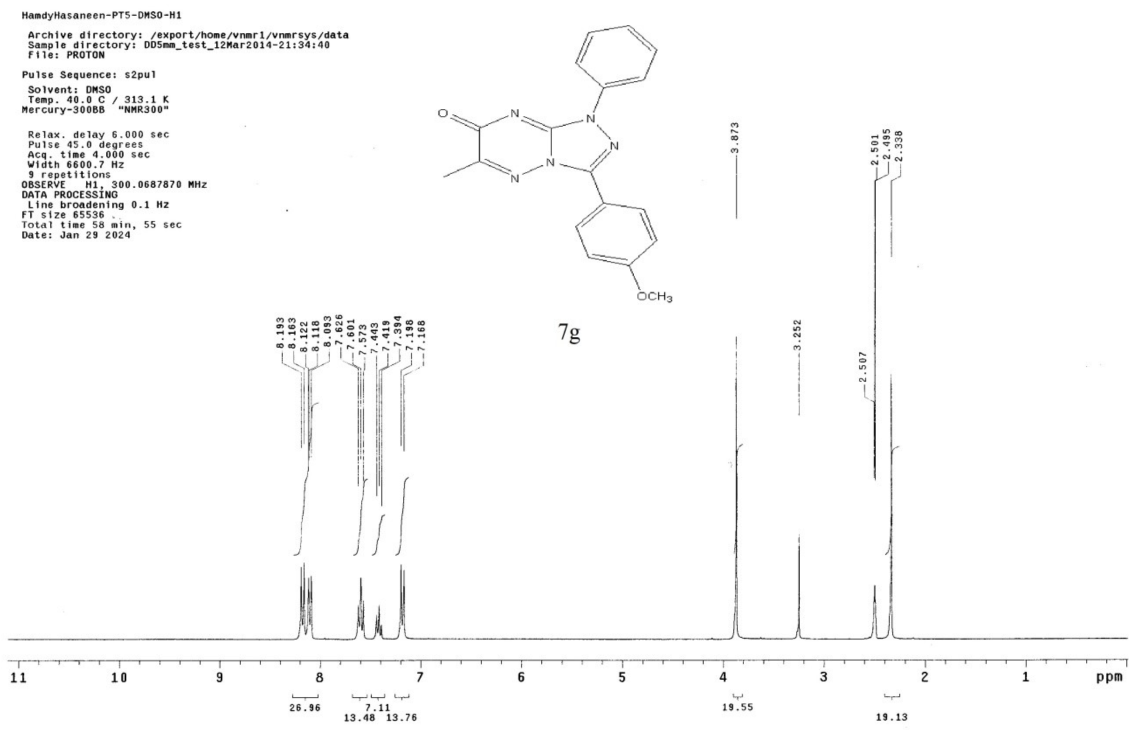

Figure S13. 1HNMR Spectrum of 7g.

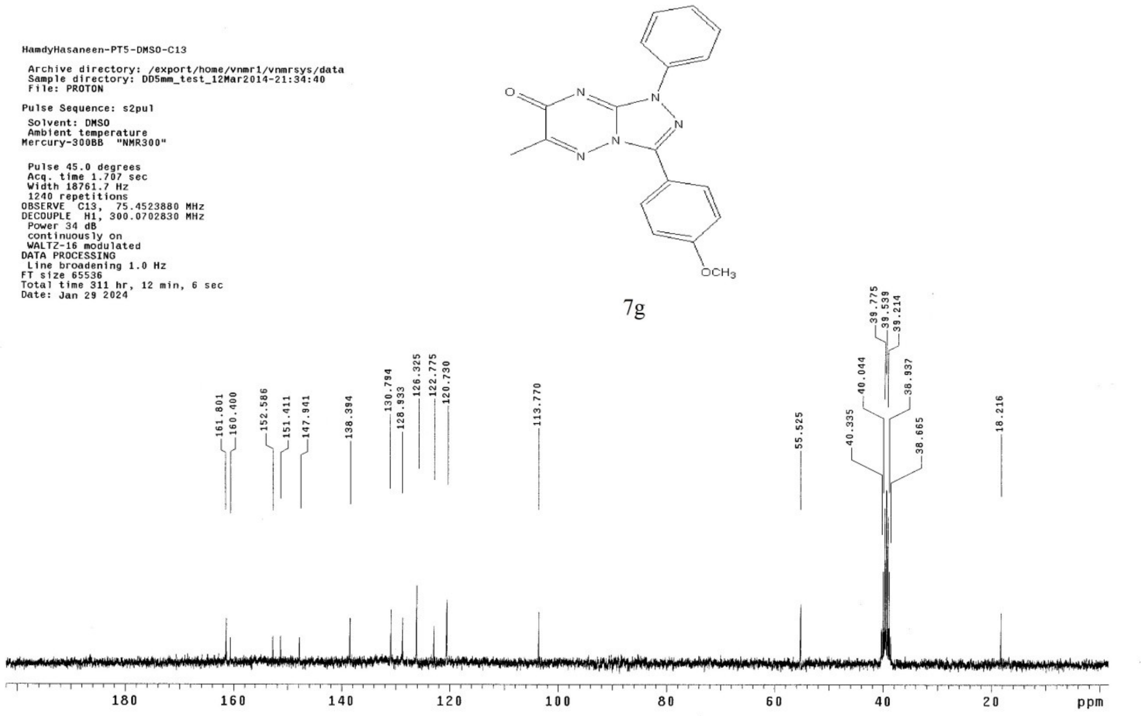

Figure S14. 13CNMR Spectrum of 7g.

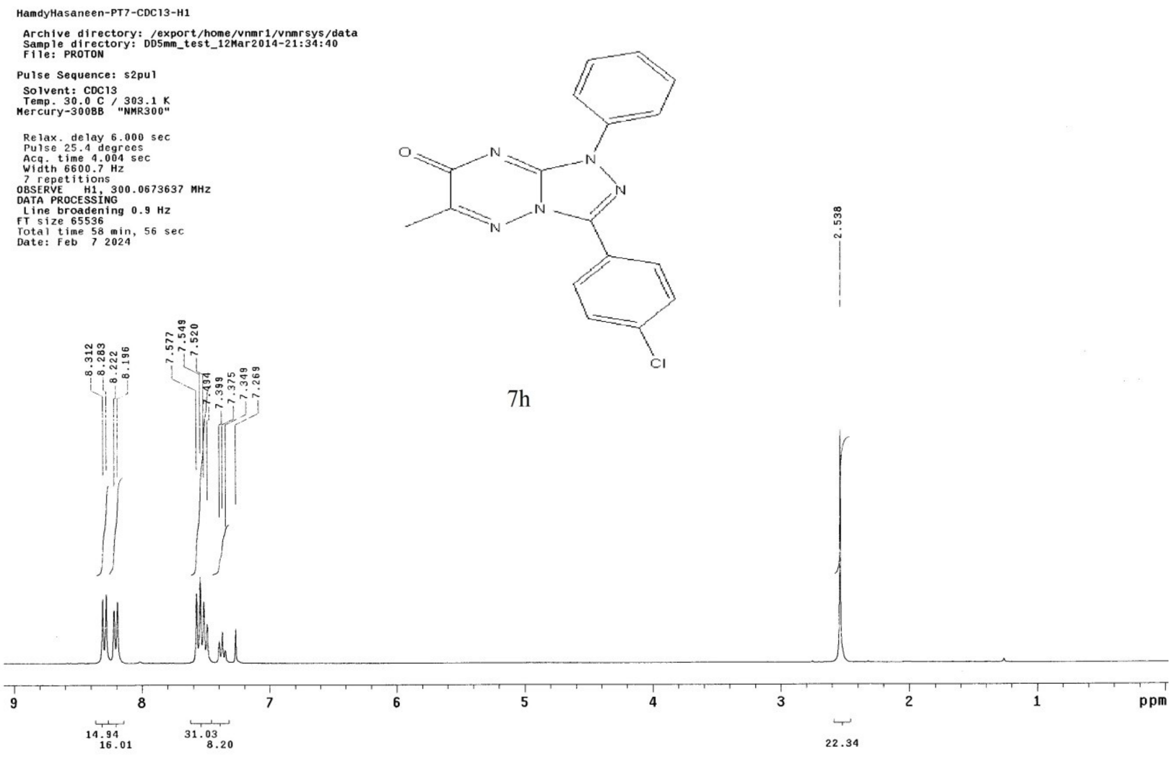

Figure S15. 1HNMR Spectrum of 7h.

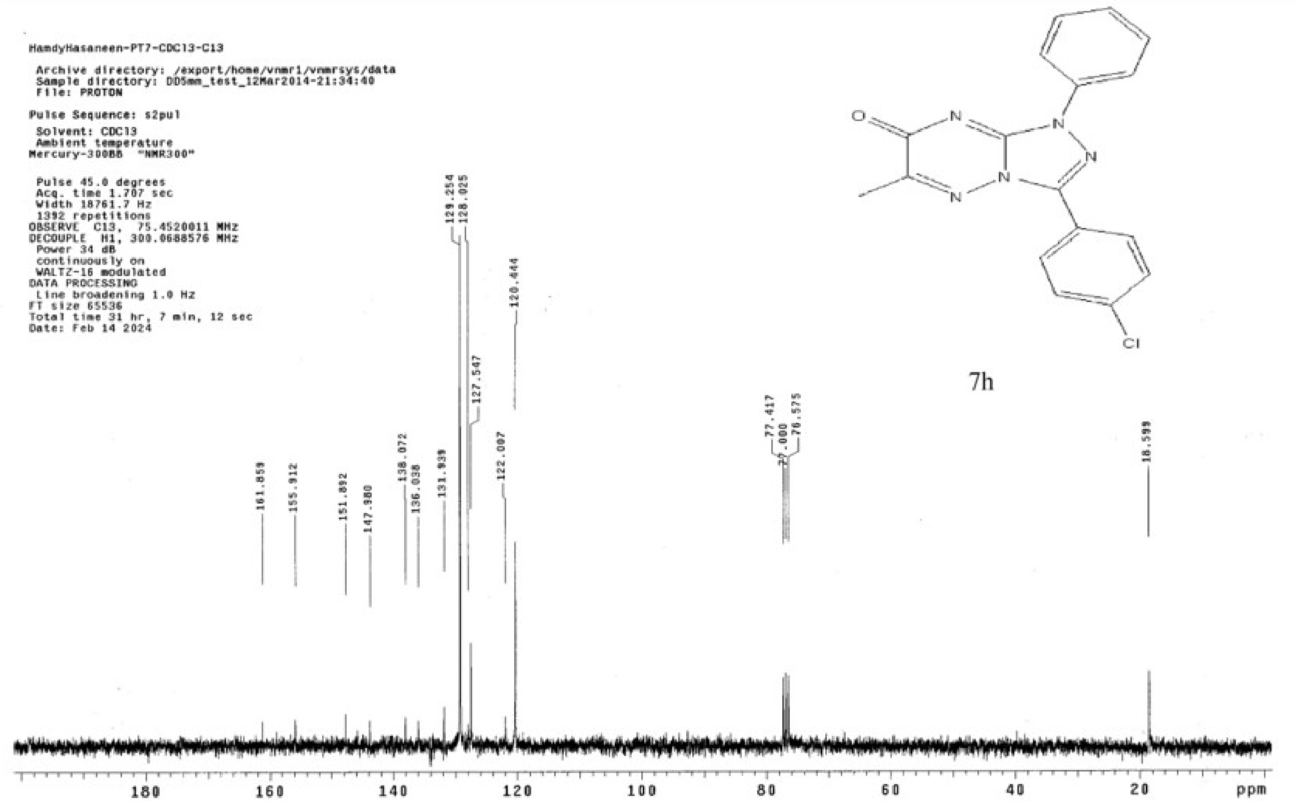

Figure S16. 13CNMR Spectrum of 7h.

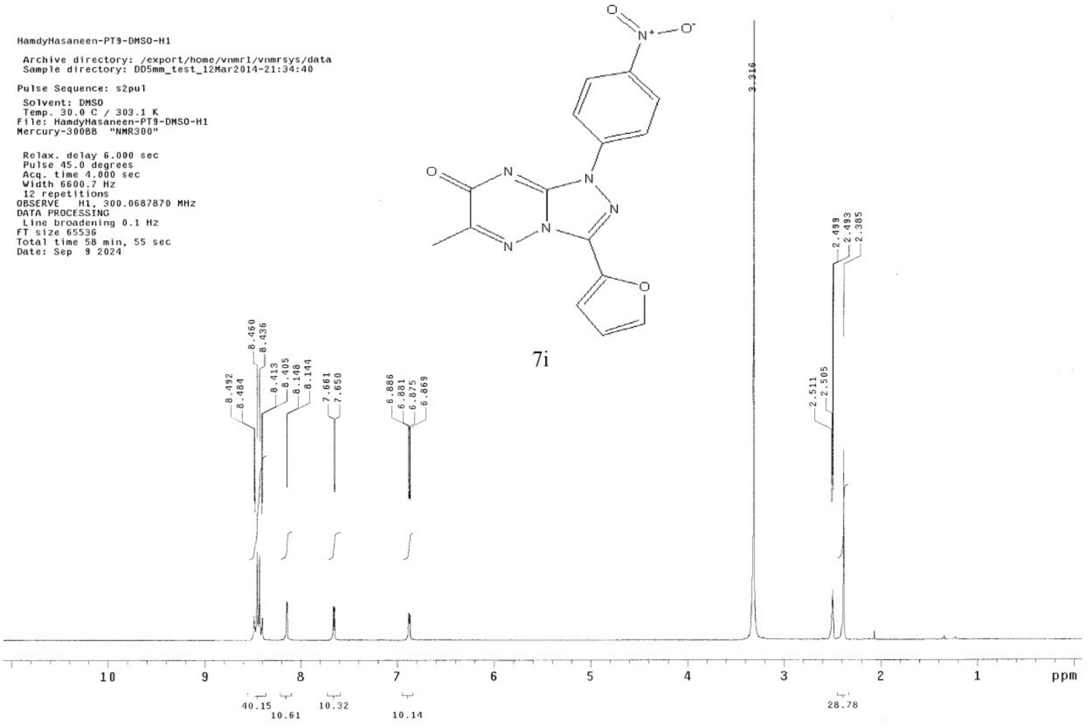

Figure S17. 1HNMR Spectrum of 7i.

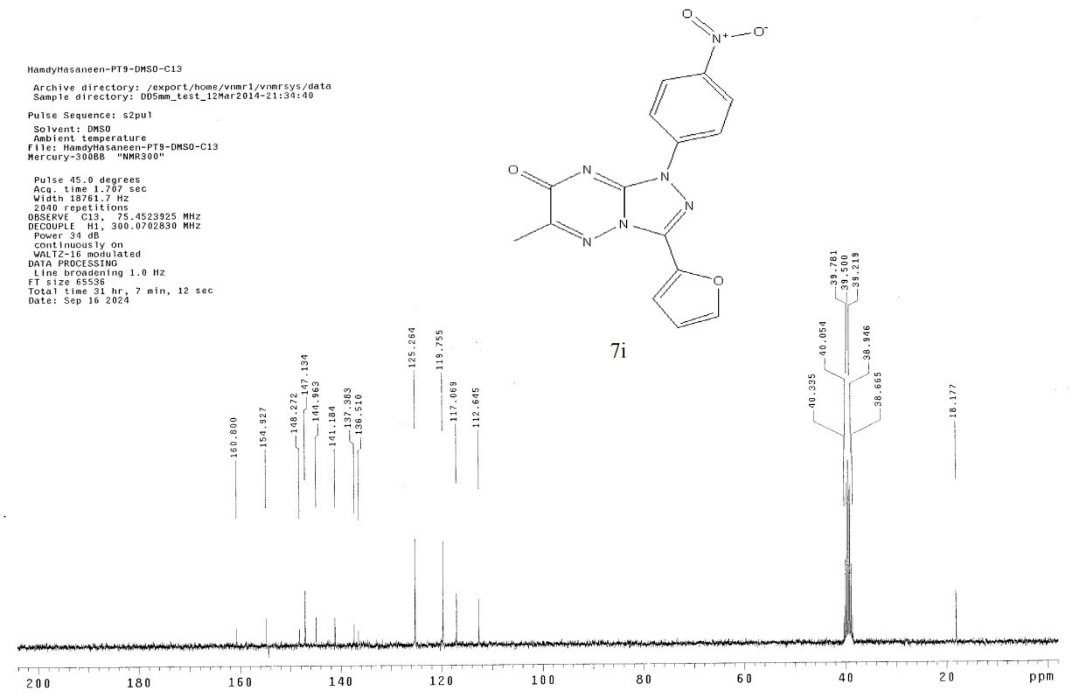

Figure S18. 13CNMR Spectrum of 7i.

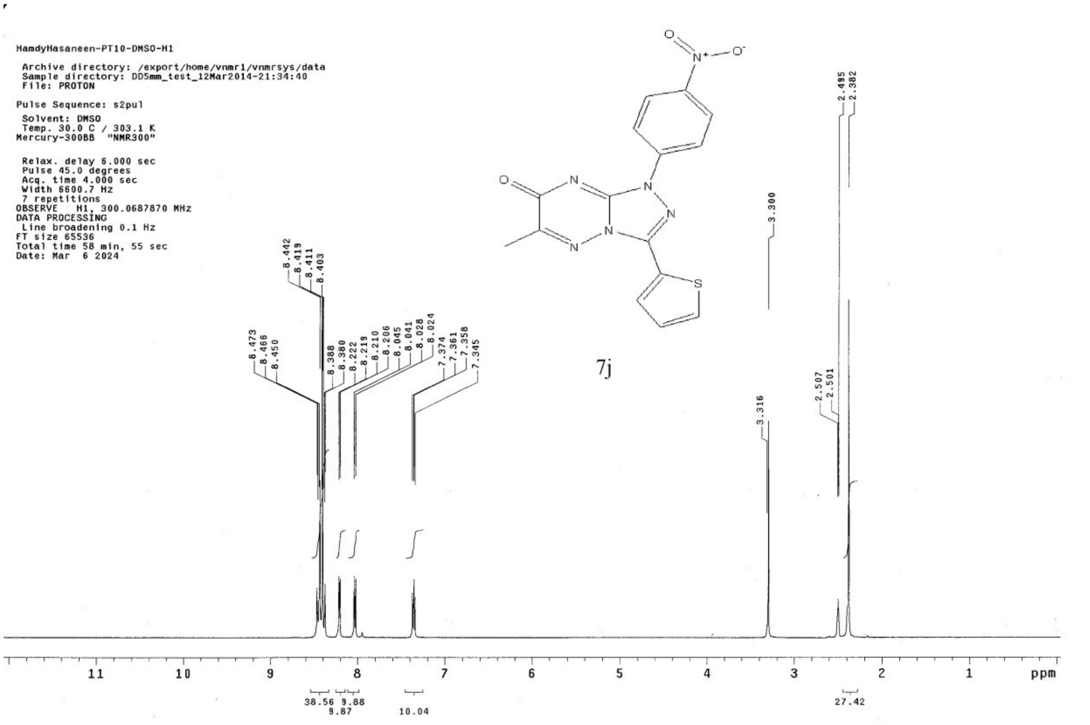

Figure S19. 1HNMR Spectrum of 7j.

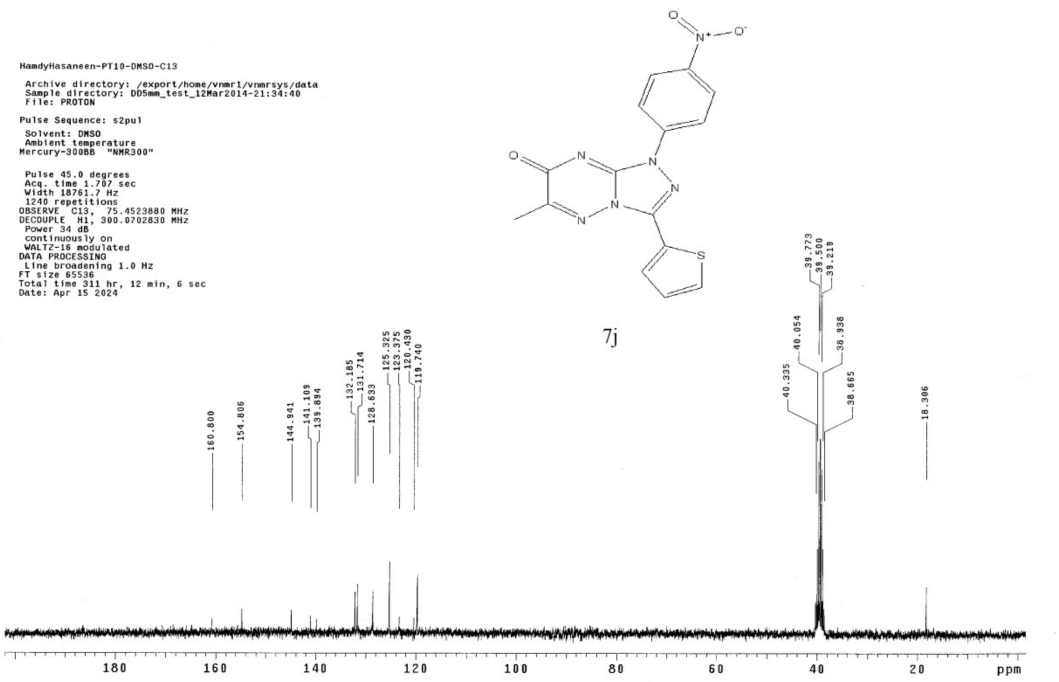

Figure S20. 13CNMR Spectrum of 7j.

Crystal data

$C_{13}H_{13}N_6O_3$	
M_r = 301.28	D_x = 1.516 Mg m^{-3}
Monoclinic, $P2_1/n$	
Hall symbol: -P 2yn	Cu *K*α radiation, λ = 1.54184 Å
a = 8.0047 (3) Å	Cell parameters from 35544 reflections
b = 21.1874 (6) Å	θ = 4.2–79.4°
c = 8.0296 (3) Å	μ = 0.95 mm^{-1}

β = 104.314 (4)°	T = 100 K
V = 1319.53 (9) Å^3	Bloc, orange
Z = 4	0.19 × 0.18 × 0.14 mm
F(000) = 628	

Data collection

XtaLAB Synergy, Dualflex, HyPix diffractometer	2843 independent reflections
Radiation source: micro-focus sealed X-ray tube, PhotonJet (Cu) X-ray Source	2742 reflections with $I > 2.0\sigma(I)$
Mirror monochromator	R_{int} = 0.042
Detector resolution: 10.0000 pixels mm^{-1}	θ_{max} = 80.0°, θ_{min} = 4.2°
φ & ω scans	h = −9 10
Absorption correction: multi-scan CrysAlisPro 1.171.42.102a (Rigaku OD, 2023)	k = −26 24
T_{min} = 0.66, T_{max} = 0.88	l = −10 10
46395 measured reflections	

Refinement

Refinement on F^2	
Least-squares matrix: full	Hydrogen site location: difference Fourier map
$R[F^2 > 2\sigma(F^2)]$ = 0.043	H atoms treated by a mixture of independent and constrained refinement
$wR(F^2)$ = 0.129	Method = Modified Sheldrick $w = 1/[\sigma^2(F^2) + (0.09P)^2 + 0.59P]$, where $P = (\max(F_o^2,0) + 2F_c^2)/3$
S = 0.98	$(\Delta/\sigma)_{max}$ = 0.001
2840 reflections	$\Delta\rho_{max}$ = 0.29 e Å^{-3}
203 parameters	$\Delta\rho_{min}$ = −0.64 e Å^{-3}
4 restraints	Extinction correction: None

Fractional atomic coordinates and isotropic or equivalent isotropic displacement parameters

(Å^2)

	x	y	z	U_{iso}*/U_{eq}
O18	1.07357 (14)	0.76108 (5)	0.30858 (15)	0.0337
O17	1.26358 (13)	0.70709 (5)	0.49217 (13)	0.0287
N9	0.89466 (14)	0.42662 (5)	0.17510 (14)	0.0200
H9	0.959 (2)	0.4223 (7)	0.272 (2)	0.0357 (19)*
O19	0.37054 (12)	0.51166 (4)	−0.38787 (12)	0.0250
N16	1.13373 (15)	0.71084 (5)	0.37303 (14)	0.0223
N7	0.68709 (13)	0.40695 (5)	−0.05413 (13)	0.0185
C22	0.37161 (18)	0.38134 (6)	−0.45787 (18)	0.0266
H221	0.3870	0.3355	−0.4523	0.0400*
H222	0.2511	0.3916	−0.4623	0.0400*
H223	0.4011	0.3977	−0.5612	0.0400*
C5	0.48606 (16)	0.41093 (6)	−0.30241 (17)	0.0209
N6	0.58276 (13)	0.37443 (5)	−0.18793 (13)	0.0207
N3	0.60069 (14)	0.51050 (5)	−0.15524 (14)	0.0199
C20	0.83138 (18)	0.31278 (6)	0.11750 (17)	0.0240
H201	0.7337	0.2970	0.1610	0.0291*
H202	0.8294	0.2904	0.0088	0.0291*
C10	0.89840 (16)	0.54112 (6)	0.16624 (15)	0.0183
C8	0.80908 (16)	0.38157 (6)	0.08264 (16)	0.0196
C14	1.11204 (16)	0.59556 (6)	0.38163 (16)	0.0200
H14	1.2059	0.5954	0.4805	0.0242*
C12	0.90719 (17)	0.65455 (6)	0.16219 (17)	0.0215
H12	0.8635	0.6940	0.1143	0.0257*
C21	1.00038 (19)	0.29803 (6)	0.24891 (18)	0.0266
H211	1.0978	0.3100	0.2018	0.0401*
H212	1.0057	0.3219	0.3546	0.0401*
H213	1.0062	0.2527	0.2745	0.0401*
C13	1.04719 (16)	0.65215 (6)	0.30380 (16)	0.0194

C2	0.69786 (15)	0.47122 (6)	−0.04679 (16)	0.0178
C11	0.83156 (16)	0.59845 (6)	0.09136 (16)	0.0209
H11	0.7360	0.5990	−0.0061	0.0250*
C4	0.48115 (16)	0.48168 (6)	−0.28614 (16)	0.0195
N1	0.82789 (14)	0.48294 (5)	0.09526 (13)	0.0187
C15	1.03697 (16)	0.53960 (6)	0.31159 (16)	0.0194
H15	1.0796	0.5004	0.3621	0.0234*

Atomic displacement parameters (Å^2)

	U^{11}	U^{22}	U^{33}	U^{12}	U^{13}	U^{23}
O18	0.0347 (6)	0.0152 (5)	0.0438 (6)	0.0003 (4)	−0.0044 (5)	0.0026 (4)
O17	0.0286 (5)	0.0228 (5)	0.0283 (5)	−0.0035 (4)	−0.0049 (4)	−0.0024 (4)
N9	0.0241 (5)	0.0138 (5)	0.0190 (5)	0.0021 (4)	−0.0003 (4)	0.0015 (4)
O19	0.0229 (5)	0.0237 (5)	0.0246 (5)	0.0025 (3)	−0.0015 (4)	0.0025 (4)
N16	0.0239 (5)	0.0171 (5)	0.0244 (5)	−0.0013 (4)	0.0033 (4)	−0.0009 (4)
N7	0.0190 (5)	0.0151 (5)	0.0192 (5)	−0.0006 (4)	0.0005 (4)	−0.0008 (4)
C22	0.0266 (7)	0.0229 (6)	0.0257 (7)	−0.0032 (5)	−0.0020 (5)	−0.0017 (5)
C5	0.0194 (6)	0.0198 (6)	0.0219 (6)	−0.0012 (5)	0.0024 (5)	0.0000 (5)
N6	0.0202 (5)	0.0183 (5)	0.0213 (5)	−0.0028 (4)	0.0006 (4)	−0.0023 (4)
N3	0.0213 (5)	0.0172 (5)	0.0196 (5)	0.0011 (4)	0.0019 (4)	0.0009 (4)
C20	0.0300 (7)	0.0148 (6)	0.0240 (6)	−0.0015 (5)	0.0004 (5)	0.0011 (5)
C10	0.0206 (6)	0.0154 (6)	0.0184 (6)	−0.0003 (4)	0.0038 (5)	−0.0016 (4)
C8	0.0206 (6)	0.0173 (6)	0.0197 (6)	0.0014 (4)	0.0026 (5)	0.0009 (4)
C14	0.0204 (6)	0.0189 (6)	0.0194 (6)	0.0000 (4)	0.0024 (4)	−0.0006 (4)
C12	0.0243 (6)	0.0164 (6)	0.0223 (6)	0.0016 (4)	0.0031 (5)	0.0017 (4)
C21	0.0320 (7)	0.0196 (6)	0.0252 (7)	0.0043 (5)	0.0012 (5)	0.0023 (5)
C13	0.0209 (6)	0.0156 (6)	0.0212 (6)	−0.0026 (4)	0.0041 (5)	−0.0025 (4)
C2	0.0185 (6)	0.0148 (6)	0.0196 (6)	−0.0008 (4)	0.0036 (5)	−0.0011 (4)
C11	0.0232 (6)	0.0174 (6)	0.0194 (6)	0.0010 (5)	0.0002 (5)	0.0007 (4)
C4	0.0193 (6)	0.0192 (6)	0.0192 (6)	−0.0003 (4)	0.0033 (5)	0.0008 (4)

N1	0.0221 (5)	0.0132 (5)	0.0188 (5)	0.0013 (4)	0.0012 (4)	0.0011 (4)
C15	0.0214 (6)	0.0154 (6)	0.0199 (6)	0.0013 (4)	0.0025 (5)	0.0010 (4)

Geometric parameters (Å, °)

O18—N16	1.2285 (15)	C20—H202	0.989
O17—N16	1.2289 (15)	C20—C8	1.4865 (17)
N9—H9	0.826 (14)	C20—C21	1.5282 (19)
N9—C8	1.2962 (16)	C10—C11	1.4015 (16)
N9—N1	1.3969 (14)	C10—N1	1.4156 (15)
O19—C4	1.2236 (16)	C10—C15	1.3976 (17)
N16—C13	1.4646 (15)	C14—H14	0.949
N7—N6	1.3721 (14)	C14—C13	1.3917 (17)
N7—C8	1.3851 (15)	C14—C15	1.3841 (17)
N7—C2	1.3648 (16)	C12—H12	0.950
C22—H221	0.979	C12—C13	1.3861 (17)
C22—H222	0.981	C12—C11	1.3901 (17)
C22—H223	0.980	C21—H211	0.980
C22—C5	1.4916 (17)	C21—H212	0.980
C5—N6	1.3001 (16)	C21—H213	0.980
C5—C4	1.5061 (18)	C2—N1	1.3634 (15)
N3—C2	1.3119 (16)	C11—H11	0.950
N3—C4	1.3769 (16)	C15—H15	0.950
C20—H201	0.990		
H9—N9—C8	125.2 (10)	N7—C8—N9	109.72 (11)
H9—N9—N1	127.4 (10)	H14—C14—C13	120.7
C8—N9—N1	106.15 (10)	H14—C14—C15	120.7
O17—N16—O18	123.50 (11)	C13—C14—C15	118.63 (11)
O17—N16—C13	118.04 (11)	H12—C12—C13	120.4
O18—N16—C13	118.45 (11)	H12—C12—C11	120.5
N6—N7—C8	126.81 (11)	C13—C12—C11	119.09 (11)
N6—N7—C2	123.68 (11)	C20—C21—H211	109.5

C8—N7—C2	109.18 (10)	C20—C21—H212	109.5
H221—C22—H222	109.5	H211—C21—H212	109.5
H221—C22—H223	109.5	C20—C21—H213	109.4
H222—C22—H223	109.4	H211—C21—H213	109.4
H221—C22—C5	109.6	H212—C21—H213	109.5
H222—C22—C5	109.4	N16—C13—C14	118.18 (11)
H223—C22—C5	109.5	N16—C13—C12	119.39 (11)
C22—C5—N6	118.48 (12)	C14—C13—C12	122.42 (11)
C22—C5—C4	117.84 (11)	N7—C2—N3	125.70 (11)
N6—C5—C4	123.67 (11)	N7—C2—N1	104.18 (10)
N7—N6—C5	113.31 (11)	N3—C2—N1	130.12 (11)
C2—N3—C4	114.30 (11)	C10—C11—C12	118.95 (11)
H201—C20—H202	108.0	C10—C11—H11	120.5
H201—C20—C8	109.2	C12—C11—H11	120.5
H202—C20—C8	109.1	C5—C4—N3	118.65 (11)
H201—C20—C21	109.2	C5—C4—O19	119.26 (11)
H202—C20—C21	109.2	N3—C4—O19	122.08 (12)
C8—C20—C21	112.03 (11)	C10—N1—N9	119.34 (10)
C11—C10—N1	120.67 (11)	C10—N1—C2	129.89 (10)
C11—C10—C15	121.21 (11)	N9—N1—C2	110.77 (10)
N1—C10—C15	118.12 (11)	C10—C15—C14	119.66 (11)
C20—C8—N7	123.94 (11)	C10—C15—H15	120.3
C20—C8—N9	126.32 (11)	C14—C15—H15	120.1
